# Using a ‘Students as Partners’ model to develop an authentic assessment promoting employability skills in undergraduate life science education

**DOI:** 10.1002/2211-5463.13941

**Published:** 2024-12-05

**Authors:** Kelsey Van, Sana Tasawar, Elaina B. K. Brendel, Camille Law, Anisha Mahajan, Carissa Brownell‐Riddell, Natalia Diamond, Kerry Ritchie, Jennifer M. Monk

**Affiliations:** ^1^ Department of Human Health and Nutritional Sciences University of Guelph Canada

**Keywords:** authentic assessment, critical thinking, employability skills, professional development, scientific literacy, students as partners

## Abstract

Authentic assessments (AA) include three principles, realism, cognitive challenge, and evaluative judgment, and replicate professional workplace expectations. Developing AA in undergraduate life science education is necessary to promote critical skill development and adequately prepare students for the workplace. Using a ‘Students‐as‐Partners’ (SAP) approach, five students, an educational developer and the instructor codeveloped an AA requiring students to utilize scientific literacy (SL) and critical thinking (CT) skills to develop a data extraction table and generate communication outputs for scientific and nonscientific audiences. Subsequently, the SAP‐developed AA was completed by students (*n* = 173) enrolled in a fourth‐year life sciences and pathophysiology course who completed an online survey providing feedback about their perceived development of critical skills and the relevance of the assignment to the workplace. The top transferable skills students reported the greatest growth in were SL (41.6%, *n* = 72), communication (34.7%, *n* = 60), CT (16.2%, *n* = 28), and problem‐solving (7.5%, *n* = 13). Student self‐assessed and instructor‐assessed grades were positively correlated, wherein 60.6% of students assessed their AA grades below the instructor's assessment and 4.7% of students assigned themselves the same grade as the instructor. Students' perceived stress levels were (a) negatively correlated with assignment grades and feelings of enjoyment, hope and pride, and (b) positively correlated with feelings of anger, anxiety, shame, and hopelessness while working on the assignment. This study demonstrates the impact of AA on the student learning experience and the relevance of AA to help prepare students for life science careers.

## Graphical abstract

Five students, one instructor, and one educational developer used a ‘Students‐as‐Partners’ approach to codevelop an authentic assessment intended to promote development of critical employability skills in life science undergraduate education. The assessment was completed by *n* = 173 students, who reported the greatest employability skill growth in scientific literacy (41.6%, *n* = 72), communication (34.7%, *n* = 60), critical thinking (16.2%, *n* = 28), and problem‐solving (7.5%, *n* = 13).

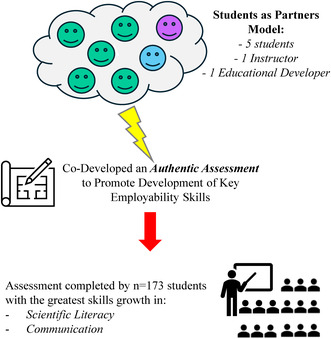

**Abbreviations**
AA: authentic assessment
AEQ‐S: shortened achievement emotion questionnaire
CT: critical thinking
PSS: perceived stress scale
SAP: students as partners
SL: scientific literacy
STEM: science, technology, engineering, and mathematics

Employability has been defined as having the knowledge, personal attributes, transferable skills, and adaptability that make individuals more likely to gain employment and be successful in their profession [1, 2]. Despite satisfying the requirements of academic programs, recent graduates do not always possess the key skills that employers expect upon entering the workplace, which adversely impacts their employment outcomes [3, 4, 5]. Compared with other disciplines, science, technology, engineering, and mathematics (STEM) graduates have been shown to take longer to find full‐time employment, in part, because of limited development of critical employability skills, such as communication, problem‐solving, critical thinking (CT), collaboration, leadership, and autonomy [6, 7, 8]. These findings indicate that improving the current educational framework to better prepare undergraduate students for the workplace environment would be beneficial [9, 10].

To support students in developing necessary employability skills, programs should consider incorporating evidence‐informed educational strategies into STEM disciplines [11, 12], wherein assessments can help to improve academic achievement, employability skill development, and enhance student learning [13, 14, 15]. In this connection, approaches proposed to support student success include, but are not limited to, case‐based learning, community‐based learning, experiential learning, and authentic assessment (AA) [11, 12, 16]. AAs differ from traditional test‐based assessment by emphasizing the application of problem‐solving and CT skills [17, 18]. Further, AA provides students with the opportunity to practice and refine competencies relevant to workplace settings [19, 20, 21] while facilitating student engagement [22] and collaboration [21]. There are three principles of an AA: (a) realism (linking course knowledge to everyday life), (b) cognitive challenge (higher order thinking and problem‐solving), and (c) evaluative judgment (performance self‐evaluation using clear criteria and receiving feedback) [17, 23]. Thus, AAs have the potential to increase the development of necessary employability skills [24, 25].

Lower levels of student engagement have been reported in STEM [26, 27, 28], and engaging students is a common challenge for educators across disciplines [29]. The development of assessments that promote engagement are valuable for student learning by encouraging CT and improving academic achievement [30, 31]. AAs can help to connect the learning outcomes of assessments to skills that are relevant for the workplace, including evaluative judgment skills [19, 32]. Another way to promote student engagement is by incorporating students' perspectives on the relevance of the assessment, which can help guide teaching approaches [33, 34, 35]. Furthermore, students do not always understand the purpose behind an assessment [32], but incorporating AA principles into an assessment can help ensure that students understand the relevance of that assessment to the development of their future employability skills, which can promote learning engagement [32, 36]. A Students‐as‐Partners (SAP) model represents one approach to improve the educational experience for students, as this model captures the student perspective while instructors and students work collaboratively to codevelop assessment strategies that include valuable student‐centered feedback [37, 38]. SAP can foster student engagement by promoting partnership between the instructor and the learner [39, 40]. Furthermore, inclusion of the student voice during assessment development can also ensure that assessments are both relevant and engaging for students [41, 42], which increases the realism AA principle. Therefore, there is a synergy between SAP and AA with the potential to increase students engagement with the assessments in their courses that could lead to increased employability skill development [17, 18, 19, 20, 21, 22], although this has not been directly evaluated. Lower levels of student engagement have been associated with academic burnout and stress [43], which can also have negative effects on students' future workplace readiness [44]. Increasing student engagement has been previously shown to mitigate the consequences of academic burnout [45], while positively impacting academic performance, educational outcomes, and students' personal development in higher education [46, 47, 48, 49]. In this connection, higher stress levels can negatively impact students' learning [50], academic performance [51, 52, 53, 54], and overall well‐being [55]. The perceived stress scale (PSS) is a validated assessment tool that measures an individuals perceived stress experienced from all sources (i.e., both academic and nonacademic sources of stress) [56], wherein higher PSS scores have been shown to negatively impact scientific literacy (SL) skill development [57] and academic performance [54]. Apart from stress, other emotions students experience while learning, both positive (e.g., enjoyment, hope, and pride) and negative (e.g., anger, anxiety, shame, hopelessness, and boredom), as assessed in the Achievement Emotions Questionnaire [58], can affect their engagement, academic achievement, and SL skill development [57, 59], which is one example of a critical STEM employability skill. Therefore, both stress and other emotional experiences can impact academic outcomes and the development of critical employability skills, which highlights the importance of using a SAP approach to better understand the student learning experience and use this critical feedback to develop an AA that can engage students in the development of critical employability skills.

To our knowledge, no studies have explored the impact of a SAP generated AA on the development of students' transferable skills for the STEM workplace. Therefore, the objectives of this study were to (a) use a SAP approach to develop a new assessment that incorporates AA dimensions in an upper‐year life science disease pathophysiology course, and (b) determine students' perspectives of the employability skills developed while completing the assessment when it was first introduced into a large‐sized undergraduate class. We hypothesized that the SAP codeveloped assessment would increase students self‐reported development of critical employability skills.

## Materials and methods

### Study overview and ethics approval

This study was conducted at a medium‐sized research‐intensive Canadian university within the context of a large‐sized (> 200 student enrollment) fourth‐year life science disease pathophysiology course that emphasized molecular and cellular mechanisms of disease progression. This study was comprised of two parts: (a) AA development using a SAP approach to codevelop an AA in collaboration with the course instructor that was completed in the summer and fall 2022 semesters, and (b) an online survey to obtain students' perceptions of the newly developed AA after it was first utilized in the course during the winter 2023 semester. Of the 208 students enrolled in the course, 173 students (83%) completed the online survey and were included in the data analysis. All participants provided their informed written consent, and this study was approved by the institutional Research Ethics Board (REB#21‐06‐022).

### Authentic assessment development using a ‘Students as Partners’ approach

The AA was called the ‘Data Extraction Assignment’ (worth 30% of the final grade), was redesigned from a preexisting assignment and was codeveloped by one course instructor, one educational developer, and five undergraduate student partners (each in the final year of their undergraduate program) that had recently completed the course in the winter 2022 semester. Student partners codeveloped the new authentic assessment in the course by providing diverse perspectives with respect to their personal and academic backgrounds, as summarized in Table 1. The SAP approach utilized student consultation and incorporated student feedback to ensure that instructor and student expectations of the assessment were aligned. The SAP approach was modeled after the design thinking process, which centers on people and their needs as the basis of any good design [60]. The collaboration between the team started near the beginning of the summer semester to develop the AA to be completed by students in the following winter semester. Therefore, throughout the summer, the team had frequent meetings to build a report, share, and understand each individual perspective contributing to the team and build empathy for each team members perspectives and experiences that informed their contribution to the project. The development of the assignment components was an iterative process wherein many possible assessment types were considered before the team centered on the Data Extraction Assignment. Therefore, the development of the Data Extraction Assignment was an iterative process that went through the following steps of the design thinking process including empathize, define, ideate, prototype, and test [60]. Student partner perspectives and input were central to the development of the assessment, and students were paid hourly for their contributions to the project. Importantly, students provided feedback about their past experiences in the course and their interpretation of the applicability of the assessment to scientific workplace settings. Additionally, student partners codeveloped the assessment instructions and rubrics to reduce ambiguities in the interpretation of the assessment expectations during the summer and early fall. Subsequently, during the fall semester, student partners created assessment exemplars that would serve as resources for future students to aid in the process of evaluative judgment of their own work by comparing completed assessment examples and rubrics to their own work. Assignment instructions and rubrics are provided in Data S1 and S2. The major student partner feedback themes collected from a written reflection exercise at the end of the project are summarized in Table 2.

**Table 1:** Student partner demographic information^a^.

Positionality	Cumulative average in undergraduate program	Final grade in the course	Challenges experienced in undergraduate assessments
Non‐Binary; International Student	62.8%	66%	Relevance of content to the real‐world
Female; 1st Generation Immigrant from an Equity Deserving Group	78.8%	82%	Memorizing content with limited relevance to real‐world
LGBTQ2S+ Non‐Binary Individual from an Equity Deserving Group	87.2%	91%	Assessments that do not adequately assess students' knowledge or provide knowledge application opportunities
Caucasian Female with Mental & Physical Health Challenges	77.0%	73%	Assignments that do not assess learning comprehension or scientific communication
Female; 2nd Generation Immigrant from an Equity Deserving Group; 1st Generation University Student	90.2%	90%	Group assignments permitting unequal workload distribution and assessments that do not permit diverse perspectives

^a^ Demographic data for the student partners (*n* = 5) that participated in the development of the data extraction assignment that had recently completed the course.

**Table 2:** Reflection themes from student partners about the SAP experience^a^.

SAP experience themes	Frequency, *n* (%)
Valued the opportunity to collaborate with peers, share perspectives and learn from other students with diverse educational and personal experiences	5 (100%)
Perceived an increase in their individual evaluative judgment skills	5 (100%)
Increased confidence in communication skills	2 (40%)
Gained a better understanding of how they prefer to have their learning and skill development assessed in courses	5 (100%)
Gained a better understanding of instructors' perspectives and the work that goes into developing assessments	3 (60%)
Felt that their feedback was included and appreciated	5 (100%)
Appreciated feedback being incorporated into the assessment instructions and rubrics	5 (100%)
Appreciated the inclusion of students' perspectives into assessment development that can benefit future students learning	5 (100%)
Recognized in the value of including different types of assessments in courses (i.e., assignments and exams)	2 (40%)
Enjoyed interacting with the instructor and learning their perspective	3 (60%)
SaP was a positive educational and personal experience	5 (100%)

^a^ Student partner (*n* = 5) reflective feedback themes about their experiences working in on assessment development using a Students as Partners (SAP) approach.

**Fig. 1:**
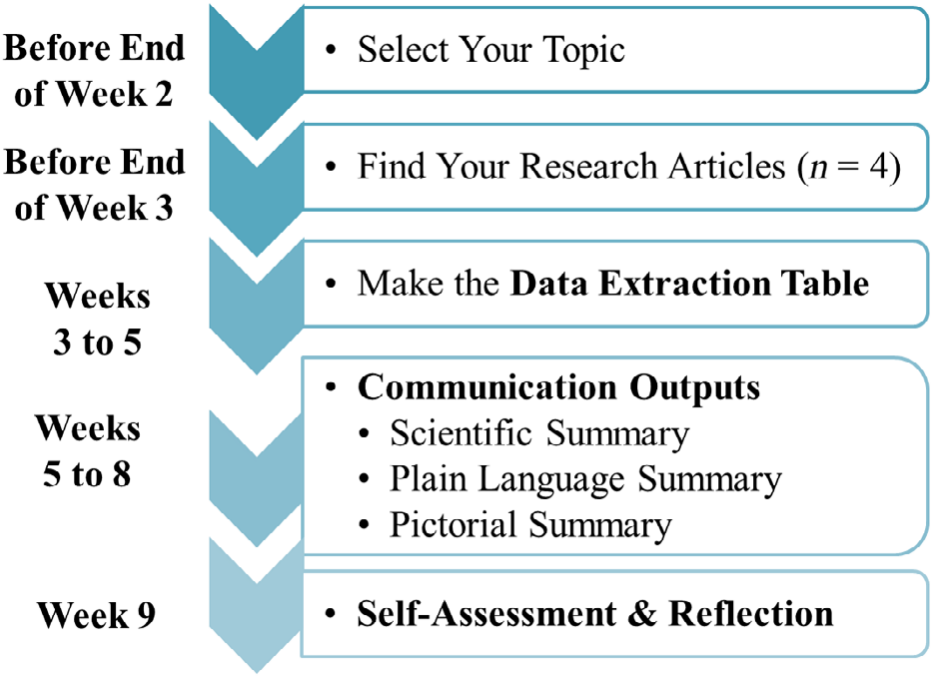
Timeline for completing the data extraction assignment components.

The resultant Data Extraction Assignment aimed to emphasize SL and CT skill development along with communication skills that are relevant for future life science careers and applicable STEM workplace tasks. These skills were in alignment with critical skills included in the course learning outcomes. Additional skills in the course learning outcomes that are related to this assignment included professional and respectful collaboration, time management, and organizational skills. The final assignment was worth 30% of students' final grade in the course and the suggested time line for completing the assignment requirements over a 9‐week period is shown in Fig. 1. Students were permitted to work alone, or in self‐selected groups of two or three students. The number of primary research articles that were summarized into the Data Extraction Table differed based on the number of students per group (i.e., four articles for students working alone, five articles for students working in groups of two, and six articles for students working in groups of three). Importantly, despite the option to work in groups with a minimal increase in workload (i.e., the addition of one research article for each additional group member), 78% of students (*n* = 135) chose to work on the assignment individually, whereas 22% (*n* = 38) chose to work on the assignment collaboratively as part of a student‐selected group. This may reflect the potential academic stress associated with group work, including workload distribution among members, discrepancies in work quality between members, the impact on grades and/or social interaction anxiety [61].

In brief, the assignment required students to select a topic related to any dietary component (nutrient, nutraceutical, or bioactive compound) and a disease of interest that was not discussed in the course. Next, students would find the appropriate number of primary research articles related to their selected topic. The selected primary articles were summarized into a data extraction table to facilitate comparisons between scientific studies (worth 30 marks). Students used the data extraction table to generate three separate communication outputs that were intended for either scientific or general audiences and mimicked the types of communication outputs students would use in the STEM workplace. These communication outputs and the marks allocated included (a) a two‐page scientific summary (intended for a scientific audience) that provided a brief background of the topic, compared and contrasted the findings from their primary articles, identified current knowledge gaps and a brief explanation of a follow‐up study that would address the identified knowledge gap to advance the research field (worth 30 marks), (b) a plain language summary (approximately 300 words and intended for a general audience) that summarized the current evidence for the effectiveness of their dietary component to modulate their selected disease of interest (as presented in their data extraction table; worth 15 marks), and (c) a pictorial summary/infographic (intended for either a scientific or general audience) that explained the mechanism of action through which the selected dietary component influenced disease severity and/or clinical outcomes (worth 15 marks). After submitting the finished assignment, each student completed a self‐assessment and reflection (worth 10 marks). For this component, the students used the assessment rubrics to grade their submitted assignment and answered a series of reflection questions (Data S1 and S2). These questions encompassed their learning experiences, challenges encountered, and strategies employed to overcome these challenges. Throughout the semester, students could request feedback on their assignment topic choice, challenges associated with the data extraction table, and/or their communication outputs through the discussion board on the course website, during weekly office hours with the instructor, or during drop‐in help sessions with the teaching assistant that were held over Zoom on a biweekly basis. Conflicts between students working in groups were minimal, likely because the group work element of the assignment was optional; however, any conflicts that arose (in two groups) were mediated by the course instructor.

### Online survey

After completing the Data Extraction Assignment, students were invited to participate in an optional online survey. As an incentive for survey participation, a 2% bonus was added to student's final examination grades. An alternate assignment was available for students who did not want to complete the online survey but still wanted to earn the participation bonus. The online survey questions included the (a) Perceived Stress Scale (PSS) [56], which is a validated 14‐item scale assessing perceived stress experienced from all sources, (b) learning‐related emotions experienced while working on the data extraction assignment using the Shortened Achievement Emotion Questionnaire (AEQ‐S) [62], which is a validated 32‐item scale (eight emotions assessed, four questions per emotion: enjoyment, hope, pride, anger, anxiety, shame, hopelessness, and boredom) that was restricted to the learning‐related questions but within the context of working on the Data Extraction Assignment, and (c) researcher generated questions to ascertain students' perceptions of the data extraction assignment, the skills students perceived they developed while working on this assignment, and the relevance of these skills to the STEM workplace.

### Statistical analysis

Statistical analyses were conducted using graphpad prism (San Diego, CA, USA) with a predefined upper limit of probability for statistical significance was *P* < 0.05. Values are presented as means ± SEM. Pearson's correlation analyses were conducted to determine the relationships between parameters.

## Results

### Students' self‐assessed assignment grades tend to be lower than the instructors' grade assessment

**Fig. 2:**
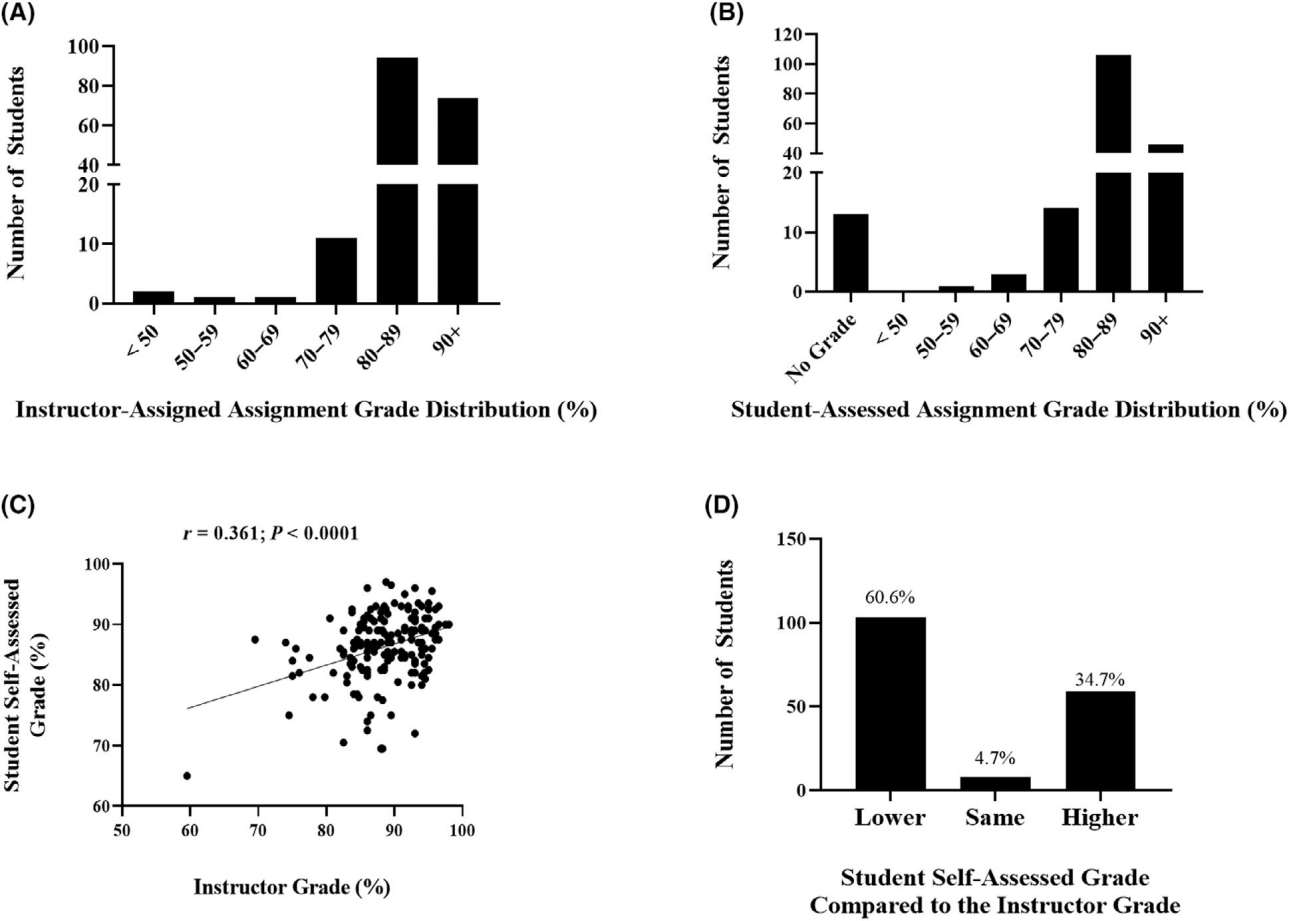
Data Extraction Assignment grade (as a %) distribution. (A) instructor‐assigned grades, (B) student self‐assessed grades, (C) relationship between students' self‐assessed assignment grade and instructor‐assessed assignment grades, and (D) distribution of students' self‐assessed grades below, the same as, or above the instructor‐assessed grade. Pearson correlation was used to determine the relationship between student and instructor assignment grades (in panel C) and the correlation coefficient (*r*) and corresponding *P* value are shown.

As a component of the evaluative judgment dimension of the data extraction assignment, students were required to use the evaluation rubrics to grade their final submitted work. The average student‐assigned data extraction assignment grade was 85.9%, and the average instructor‐assigned grade was 87.1%. The distribution of students' data extraction assignment grades based on the instructor assessment and the students' self‐assessed grades are shown in Fig. 2A,B, respectively. There was a moderate significant correlation between students' self‐assessed assignment grades and the instructor grades (*r* = 0.361; *P* < 0.001), as shown in Fig. 2C. The distribution of students' self‐assessed grades compared with the instructor's assessment is shown in Fig. 2D, wherein only 4.7% of students self‐assessed grade was in alignment with the instructor assessment. Most students (60.6%) self‐assessed grade was lower than the instructor‐assessed grade and 34.7% of students provided a self‐assessed grade that was higher than the instructor's assessment.

### Students' stress levels and negative learning‐related emotions are associated with lower academic outcomes

**Fig. 3:**
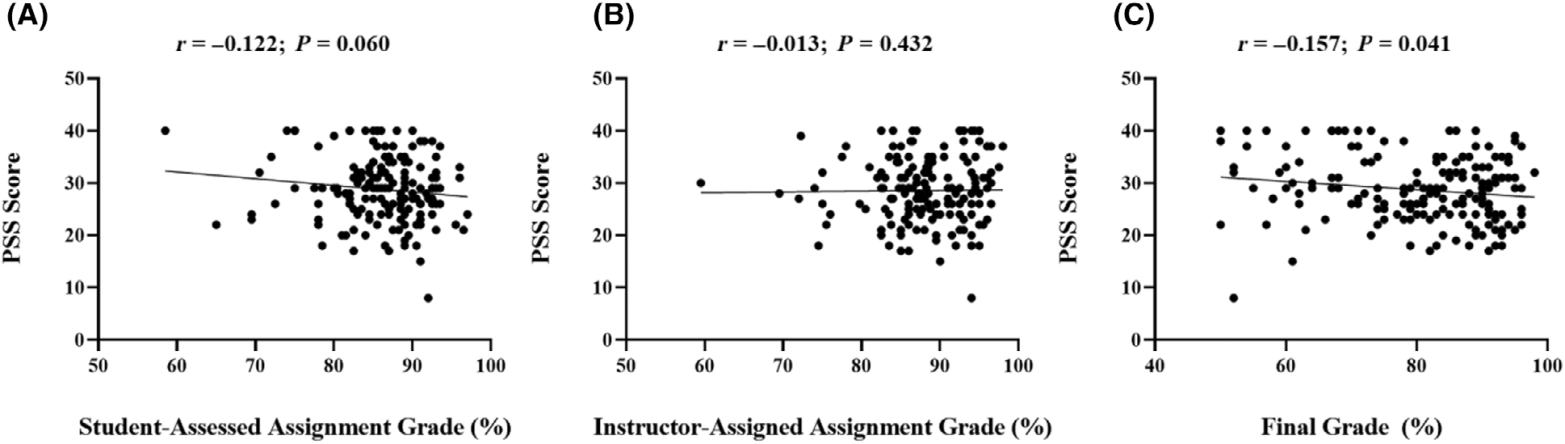
Relationship between students perceived stress scale (PSS) scores and academic performance outcome. (A) data extraction assignment student self‐assessed grade (as a %), (B) data extraction assignment instructor‐assessed grade (as a %), and (C) final grade in the course (as a %). Pearson correlation analyses were used to determine the relationship between PSS scores and academic outcomes with the correlation coefficient (*r*) and corresponding *P* value shown.

The average perceived stress levels reported by students, as assessed using the PSS, was 28.7 out of 40, which is within the highest category of perceived stress (i.e., scores 27–40) [63]. There was no relationship between students' PSS scores and either student‐assessed assignment grade or the instructor‐assessed assignment grade (*P* > 0.05; Fig. 3A,B). Conversely, PSS scores were negatively associated with students' final grade in the course (*P* < 0.05; Fig. 3C), indicating that students experiencing higher stress levels had lower overall academic achievement in the course.

Learning‐related achievement emotions (both of a positive or negative nature) are experienced during learning‐associated activities [58] and can impact both student engagement and academic achievement [59]. The relationship between students' learning‐related achievement emotions and both PSS scores and academic performance outcomes are shown in Table 3. All three positive learning‐related emotions (enjoyment, hope, and pride) were negatively correlated with PSS scores (*P* < 0.05), indicating that students experiencing higher perceived stress levels had less enjoyment, hope, or pride associated with completing the data extraction assignment. Conversely, there were positive correlations between students' PSS scores and experiencing anger, anxiety, shame, and hopelessness while working on the data extraction assignment (*P* < 0.05), indicating that the learning experience was emotionally challenging for students experiencing higher stress levels. There was no relationship between students experience of boredom and their PSS scores (*P* > 0.05).

**Table 3:** Correlations between students' learning‐related achievement emotions, perceived stress scores and academic outcomes^a^.

Learning‐related achievement emotion	Perceived stress scores (PSS)		Student self‐assessed assignment grade (%)		Instructor‐assessed assignment grade (%)		Final grade in the course (%)	
	*r*	*P*	*r*	*P*	*r*	*P*	*r*	*P*
Enjoyment	−0.126	0.050*	0.014	0.433	−0.177	0.011*	0.038	0.312
Hope	−0.285	< 0.001*	0.084	0.147	−0.100	0.098	0.071	0.180
Pride	−0.245	< 0.001*	0.135	0.046*	−0.088	0.127	−0.027	0.364
Anger	0.211	0.003*	0.002	0.492	0.032	0.338	0.017	0.414
Anxiety	0.355	< 0.001*	−0.063	0.216	−0.077	0.161	0.006	0.467
Shame	0.302	< 0.001*	−0.139	0.042*	−0.185	0.008*	−0.133	0.043*
Hopelessness	0.386	< 0.001*	−0.195	0.007*	−0.186	0.007*	−0.167	0.016*
Boredom	0.078	0.158	0.002	0.492	−0.017	0.414	−0.068	0.193

^a^ Pearson correlation coefficients (*r*) and *P* values are shown. Significant correlations are marked with an asterisk (*). Mean ± standard deviation (SD): enjoyment (13.9 ± 3.8); hope (13.3 ± 3.8); pride (14.8 ± 3.8); anger (10.2 ± 3.7); anxiety (12.8 ± 3.9); shame (9.5 ± 3.9); hopelessness (8.4 ± 4.0); boredom (10.3 ± 3.8); PSS (29.0 ± 6.9); student self‐assessed assignment grade (86.1 ± 5.9); instructor‐assessed assignment grade (87.3 ± 10.6); final grade in the course (80.1 ± 12.4).

The relationship between students learning‐related emotions and either their self‐assessed assignment grade or the instructor‐assessed assignment grade was less consistent between the positive or negative learning‐related emotions. Specifically, only pride was positively correlated with students' self‐assessed assignment grade (*P* < 0.05), whereas feelings of shame and hopelessness were negatively correlated with the self‐assessed assignment grade (*P* < 0.05). This indicates that students feeling a higher degree of shame or hopelessness while working on this assignment assigned themselves a lower grade when conducting a self‐assessment. These significant negative relationships for shame and hopelessness were also observed when correlated with the instructor‐assigned grade and with students' final grade in the course (*P* < 0.05; Table 3). Unexpectedly, students' experience of enjoyment was negatively correlated with the instructor‐assessed assignment grade (*P* < 0.05), indicating that students achieving higher instructor‐assessed grades were experiencing less enjoyment while working on this assignment. This could reflect the process of cross‐referencing assessment rubric requirements and integrating these details into the final assignment to achieve the highest possible grade. There were no other relationships observed between any other learning‐related emotion and either assignment grades or final grade in the course.

### SL and communication/knowledge translation were the employability skills students perceived the greatest skill competency growth in and relevance to the STEM workplace

Students identified the skills used or practiced developing when completing the components of the data extraction assignment and provided their perceptions of the relevance of these skills to the STEM workplace, as shown in Table 4. Additionally, students reported their perceptions of the main assignment‐associated skill and transferable skill that they exhibited the greatest growth in skill competency for, which is also shown in Table 4. Students were asked to provide their perception of the relevance of each assignment‐associated skill to the future STEM workplace, wherein the top three workplace relevant and/or employability skills included (a) organizing and integrating scientific information from multiple primary literature sources (80.9% of students), (b) finding and accessing primary research articles (69.4% of students), and (c) writing a plain language summary of a scientific concept for a general audience (60.7% of students). When students were asked to reflect on which assignment‐associated skill they perceived to have experienced the greatest growth in skill competency, and in general, growth in SL‐associated skills were higher than communication skills (Table 4). Organizing and integrating scientific information from multiple sources was perceived by students to be both relevant to the STEM workplace and the assignment‐associated skill they exhibited the greatest growth in competency. In terms of transferable skills, SL and communication/knowledge translation skills were identified as the most relevant skills for the workplace that were used in the assignment by 38.7% and 28.3% of students, respectively (Table 4). Students reported the greatest growth in these same transferable skills, namely SL (41.6% of students) and communication/knowledge translation (34.7% of students). Additionally, 16.2% of students identified growth in their CT capabilities and 7.5% of students identified growth in their problem‐solving skills (Table 4).

**Table 4:** Students' perceptions workplace relevance and skill competency growth associated with components of the data extraction assignment^a^.

	Relevance to the workplace % (number of students)	Greatest growth in Students' skill competency % (number of students)
Data extraction assignment components associated with SL skills^b^		
Finding and accessing primary research articles	69.4% (*n* = 120)	15.6% (*n* = 27)
Organizing and integrating scientific information from multiple primary literature sources	80.9% (*n* = 140)	36.4% (*n* = 63)
Making a data extraction table to organize results from multiple primary literature sources	37.6% (*n* = 65)	16.2% (*n* = 28)
Data extraction assignment components associated with communication skills^b^		
Writing a plain language summary of a scientific concept for a general audience	60.7% (*n* = 105)	9.8% (*n* = 17)
Writing a scientific summary for a scientific audience	52.0% (*n* = 90)	15.0% (*n* = 26)
Creating a pictorial summary of a scientific concept	40.5% (*n* = 70)	6.9% (*n* = 12)
Employability skills associated with the data extraction assignment^c^		
Scientific literacy through data analysis and interpretation	38.7% (*n* = 67)	41.6% (*n* = 72)
Communication and knowledge translation skills	28.3% (*n* = 49)	34.7% (*n* = 60)
Critical thinking	22.5% (*n* = 39)	16.2% (*n* = 28)
Problem‐solving	10.4% (*n* = 18)	7.5% (*n* = 13)

^a^ Values are presented as percentage and number of students identifying each component of the data extraction assignment or transferable skill associated with the assignment.

^b^ Students were asked to select any of the Data Extraction Assignment components associated with scientific literacy (SL) or communication skills that they perceived to be relevant for the science, technology, engineering and mathematics (STEM) workplace (*n* > 173). Conversely, students were asked to select the top (i.e., single) component within the Data Extraction Assignment associated with either SL or communication skills that they perceived to have experienced the greatest growth in their individual skill competency (*n* = 173).

^c^ Students' were asked to select the top (i.e., single) employability skill associated with the Data Extraction Assignment they perceived to be the most relevant to the STEM workplace and the skill they experienced the greatest growth in their individual skill competency.

## Discussion

In this study, a SAP approach involving students with diverse perspectives that recently completed the course codeveloped an assignment that incorporated AA principles (realism, cognitive challenge, and evaluative judgment including receiving feedback) [25]. Subsequently, the instructor/student codeveloped assessment was included in the next offering of the course and those students' perspectives of the experience completing the assessment along with the relevance of the assessment components to promote development of critical skills that are relevant for STEM workplace were assessed. Overall, this SAP partnership resulted in a meaningful and sustainable new assessment that helped develop students' SL and communication/knowledge translation skills that were also associated with increased student feelings of pride and reduced boredom and shame. Student attitudes toward learning have been shown to impact academic performance [57], which can also influence the degree in which students develop critical employability skills through assessment in higher education [64].

Using a SAP approach can be an effective tool for improving assessment methods [65, 66] and the resulting assessment product can increase student engagement [42]. The inclusion of student partner perspectives during assessment development provided an opportunity for bidirectional learning wherein (a) student partners learned about assessment authenticity principles and the intentionality through which assessments are developed to support practical and relevant skill development that can promote career readiness, and (b) instructor partners learn from students about the skills and experiences they believe are relevant. Through this student feedback, instructors can improve their communication approaches on assignment instructions to ensure that the intentions of the assessment (i.e., relevance to the workplace and promoting skill development) are phrased in a manner that resonates with students. A challenge in fostering the development of employability skills is that students do not always understand the purpose behind an assessment [32]. AA can help to ensure that students understand the relevance of an assessment to their future employability and/or role in society, which can promote a sense of purpose to support their motivation, engagement, and self‐esteem [32, 36]. Collaborating with student partners during the process of assessment design ensures that assessments are aligned with both instructor and students' goals and can increase students' perceived credibility or relevance of assignments [67]. There are many potential benefits associated with SAP collaborations [68]; however, the critical benefit that underpins the successful partnership in this study is the shared understanding of each instructor and students' perspectives and needs in education that permits the effective collaborative development of a meaningful and beneficial assessment for students' academic development, as summarized in Table 2. A SAP approach can be implemented in the redesign or redevelopment of any aspect of a course (e.g., instructional approach, course materials, assessments); however, it is important that student partner perspectives are valued, incorporated into the final product, and that an evidence‐based approach is used to assess the effectiveness of the redesigned element of the course, as in the current study.

Appropriately designed assessments can enhance the learning process by allowing students to both demonstrate skill competency and receive feedback on relevant skills [69, 70, 71]. Recently, more attention has been directed toward evaluating the quality of assessments in higher education to maximize the learning experiences and future employability of undergraduate students [17, 65, 72]. AA can address these needs in undergraduate education and have been shown to encourage deeper learning [25, 66], increase skill competency [19, 20], and facilitate students applying their knowledge to novel situations [73]. We also showed improved transferable employability skills, specifically SL and communication skills associated with the AA (Table 4). Thus, AA can help to improve the employability of students by reinforcing the knowledge, skills, and personal‐attributes necessary for the workplace [17, 24]. The principles of AA include realism, which involves making connections between knowledge with everyday situations [23, 74, 75]; and cognitive challenge, where students use higher order cognitive skills to solve problems [25]. Further, AA can enhance development of employability skills by encouraging students to use evaluative judgment, another principle of AA, which is the ability of students to assess their performance and self‐regulate their learning process using clearly articulated assessment criteria and feedback [17, 25]. To facilitate the learning experience associated with the assessment, it is recommended to provide multiple opportunities for student feedback so students use their evaluative judgment skills. In the current study, this occurred through instructor feedback/approval of assignment topics and feedback about how to improve communication of research study findings in the data extraction table when information from multiple scientific sources were integrated into one summary table. Additionally, drop‐in help sessions (online via Zoom and on the discussion board on the course website) were provided throughout the semester to support students and provide feedback through the process of completing the assignment components. A better practice for providing students with assignment feedback to support their evaluative judgment skill development (i.e., in alignment with AA principles) [17, 23] would be to provide written feedback on the assessment and permit students to implement the feedback and revise their assessments for regrading; however, this can be a challenge with limited instructor resources and/or in large class sizes.

The results from this study suggest that students were able to use their evaluative judgment skills in the context of the data extraction assignment, since there was a moderate positive correlation between students' self‐assessed assignment grades with the instructor grades. Still, most students (60.6%) had self‐assessed grades that were lower than the instructor grade, indicating that there is room for students to improve their self‐efficacy [76]. Alignment between instructor and student self‐assessments of skill development or academic performance can be variable [77, 78, 79]. Students' self‐assessments can shed light on the developmental process of skill mastery and its measurement [80], and therefore, represents an important way to engage students in critically assessing their skills and promote development of their evaluative judgment capabilities. Providing students with clear grading expectations, as in the current study, can facilitate the self‐assessment process and limit over‐ and underestimates of skill competencies [81]. In this connection, a weak correlation between instructor and students' assessments has been shown when those assessments were based on criteria outlined in a rubric [79], and in the current study (Fig. 2). Providing grading criteria can guide the process, however, best practices will include instruction provided to students on how to conduct self‐assessments to maximize the benefits to students [79]. Underestimates of skill competencies in students' self‐assessments may reflect their developmental stage and perceptions of skill mastery, as students may feel less competent than they are and provide a lower self‐assessed grade [77]. More accurate self‐assessors have been shown to be more humble and less optimistic when critically evaluating their skills, whereas overestimators are more optimistic may be less critical and realistic about their evaluation [79]. Previously, the use of AA activities has been shown to improve students' confidence [24, 82] and self‐regulation [83], which are positively associated with self‐efficacy [84]. The inclusion of students' self‐ assessment associated with AA can help promote evaluative judgment and facilitate students' engagement in critical self‐reflection that can lead to improved confidence and a better appreciation of their individual skill competencies.

A challenge in undergraduate STEM education is ensuring the development of skills necessary for employment post‐graduation [64, 72, 85]. Recent graduates' skill competencies do not necessarily satisfy employers' expectations [86, 87, 88]. Traditional test‐based assessment methods often reinforce rote memorization, which is not a workplace relevant skill [15], in contrast with SL, problem‐solving, CT and communication or knowledge translation that are desirable for future employability [85, 89, 90, 91] but frequently lacking in STEM graduates [85, 91, 92, 93]. Interestingly, students reported increasing their competency in these same workplace relevant transferable skills in association with the data extraction assignment (Table 4). Amongst the skills that were improved, increased SL skills was the top skill developed by 41.6% of students. In contrast, problem‐solving skills were improved in only 7.5% of students. The increase in SL skills is expected in an assignment centered on accessing, interpreting, and integrating scientific results from multiple studies in the process of generating a data extraction table and then using the table to create different forms of communication outputs. In this connection, improved communication/knowledge translation skills was the second most commonly improved skill associated with the AA (34.7% of students). Interestingly, problem‐solving skills were perceived to be improved by only 7.5% of students. Although students would have utilized problem‐solving skills to overcome any challenges they encountered while working on the assignment, the value of these skill developing experiences and/or the use of problem‐solving skills were not recognized by many students. Problem‐solving skill competency is a critical employability skills for STEM graduates [94, 95, 96, 97, 98, 99, 100] that is lacking amongst STEM [101] and life sciences professional (e.g., nursing) [102, 103, 104] graduates. Transferable skills, such as problem‐solving skills, may be developed in association with various assessments and educational experiences; however, they are not always explicitly highlighted or discussed with students in contrast to discipline‐specific or technical skills [105]. Therefore, problem‐solving skills and/or other types of transferable skills were implicit and may have been developed in association with this assignment, in contrast to the highlighted STEM‐associated skills developed or practiced through the Data Extraction Assignment. Studies have highlighted emphasis placed on discipline‐specific or technical employability skills over transferable skills and the implicit assumption that students will pursue careers directly related to their degree subject [106, 107]. Future iterations of this assignment should explicitly discuss and differentiate between discipline‐specific and transferable skills and include a reflection question surrounding students' use of these two categories of skills to help broaden students' perspectives of employability skills that they are developing while working on this assignment. Student reflection on the types of skills developed in association with an assessment (as required in the Data Extraction Assignment) is highly relevant, as exercising awareness of transferable skill development in relation to future employability has been established [42]. Collectively, these data support the need to redevelop assessments in higher education to promote the development of job readiness skills [108, 109, 110].

There is considerable evidence indicating that self‐efficacy is a strong predictor of academic achievement [111, 112], which is defined as an individual's belief in their own ability to achieve a desired outcome [113]. Self‐efficacy can explain individual differences in motivation and attitude when learning [76], and impacts students' metacognition, strategies, and learning‐related emotions to influence academic performance [84, 114, 115]. Increasing self‐efficacy has been identified as an important factor to mitigate higher levels of stress and depression in students [84, 116, 117, 118]. Therefore, approaches to attenuate levels of stress in university students should focus on increasing students' self‐efficacy [119], which can be improved, in part, through self‐evaluation and increased evaluative judgment skills, a key principle of AA. Higher stress levels can negatively impact students' academic performance [51, 53], learning process [50], and overall well‐being [55]. PSS scores reflect the magnitude of stress experienced by students from all sources (both academic and nonacademic sources), which can impact learning engagement and academic performance. The negative association between students' final grades and PSS scores (Fig. 3) in this study confirm previous research demonstrating that higher stress levels can negatively influence academic achievement [51, 52, 53]. Students' self‐assessed and instructor‐assessed assignment grades were not related to their PSS scores (Fig. 3). Conversely, PSS scores were negatively correlated with positive learning‐related emotions, while PSS scores were concomitantly positively correlated with negative learning‐related emotions (Table 3). Higher levels of stress can also increase the incidence of depression [84, 120, 121] and hinder the well‐being of students [55]. For these reasons, it is imperative to utilize assessment approaches to reduce levels of stress in students to improve their academic performance and overall well‐being [55, 122, 123]. Not unsurprisingly, students' stress experience is also related to their experience of other learning‐related emotions including positive learning‐related emotions (enjoyment, hope, and pride) negative learning‐related emotions (anger, anxiety, shame, hopelessness, and boredom). Previous studies have shown that students experiencing higher perceived stress levels also experience more negative and fewer positive learning‐related emotions [57], and these relationships were also observed in the current study (Table 3). Therefore, the data underscore the relationships between students' stress and emotional experiences and academic performance. Positive learning‐related emotions have been shown to positively influence academic achievement, whereas negative learning‐related emotions can reduce motivation and negatively affect academic achievement [57], as also shown in the current study (Table 3). Furthermore, the influence of learning‐related emotions on students' perceptions of SL skill competency exhibited a similar relationship, wherein students experiencing positive learning‐related emotions had increased perceived SL capabilities, whereas those experiencing more negative learning‐related emotions had lower perceptions of their SL skill capabilities [57], although the inclusion of AA principles were not evaluated in this study. Taken together, these results not only provide support for the relationship between stress and learning emotions to impact academic outcomes but also highlight the relationship between students' emotional experience while learning or completing course assessments (beyond their stress experience) and their academic performance and/or skill development. The current study did not evaluate the relationship between students perceived employability skill development and their emotional experience; however, future studies should evaluate how students' attitudes, emotional experience and stress experience while learning impacts their employability skill development (both perceived capabilities and practical skill capabilities). Gaining insight into these elements of the student learning experience through a SAP approach could further help inform the development of an AA that promotes employability skill development, particularly when an evaluation tool is used to determine an assessments inclusion of authenticity principles [17, 18].

This study was centered on a specific assignment codeveloped by student partners and the course instructor, and therefore, may be interpreted to have limited applicability to other courses, year of university study, and discipline contexts. However, the core skills associated with the assignment, communication [94, 124], knowledge translation [125, 126, 127], CT [128, 129], and SL [130, 131], are highly relevant across disciplines and course contexts to prepare students for the STEM workplace. The assessment of skill development was primarily based on students' self‐evaluation and perceptions, rather than a practical skill assessment conducted before and after completion of the assignment; therefore, future studies should assess the influence of an AA on practical skill growth. The current study did not include a control group that assessed students' employability skill perceptions without engagement in the Data Extraction Assignment, which is a limitation in the study design. Finally, students' perceived skill development cannot be exclusively attributed to this assessment as other educational experiences in other courses were simultaneously occurring. Despite these limitations, students' perceived skill development remains a viable outcome measure [132, 133, 134]. Furthermore, the ability for students to relate the skills developed and/or practiced in association with the Data Extraction Assignment demonstrates, in part, that the AA principles were achieved by connecting assignment components to the future workplace [32].

AA should be used to improve rather than replace existing assessments, and through this approach improve educational experiences in higher education [25]. The current study demonstrated the use of a SAP approach to collaboratively develop an AA that impacted student perceptions of their critical employability skills that are relevant for the STEM workplace [32]. Although the assignment resources (instructions and rubrics) were developed in the context of a life sciences and pathophysiology course, these materials could be adapted to other STEM course contexts, which enhances the applicability of this work to other disciplines.

## Conflict of interest

The authors declare no conflict of interest.

## Author contributions

JMM and KR conceived and designed the project. KV, CB‐R, ND, EBKB, CL, AM, and JMM acquired, analyzed, and interpreted the data. CL, EBKB, and ND prepared the figures and tables. KV and JMM wrote the paper. All authors edited and approved the final paper. KR and JMM acquired financial support for the project.

## Supporting information


**Data S1.** Data extraction assignment instructions.

**Data S2.** Data extraction assignment self‐assessment template, rubrics and reflection questions.

## Data Availability

The data that support the findings of this study are available in Figs 2 and 3, Tables 3 and 4 of this article.

## References

[1] Small L , Shacklock K and Marchant T (2017) Employability: a contemporary review for higher education stakeholders. J Vocat Educ Train 70, 1–19.

[2] Williams S , Karypidou A , Steele C and Dodd L (2019) A personal construct approach to employability: comparing stakeholders' implicit theories. Educ Train 61, 390–412.

[3] Tushar H and Sooraksa N (2023) Global employability skills in the 21st century workplace: a semi‐systematic literature review. Heliyon 9, e21023. 37954286 10.1016/j.heliyon.2023.e21023 PMC10637906

[4] Clarke M (2018) Rethinking graduate employability: the role of capital, individual attributes and context. Stud High Educ 43, 1923–1937.

[5] Cheng M , Adekola O , Albia J and Cai S (2022) Employability in higher education: a review of key stakeholders' perspectives. High Educ Eval Dev 16, 16–31.

[6] Aldriwesh MG , Alyousif SM and Alharbi NS (2022) Undergraduate‐level teaching and learning approaches for interprofessional education in the health professions: a systematic review. BMC Med Educ 22, 13. 34980083 10.1186/s12909-021-03073-0 PMC8725543

[7] Singh P , Thambusamy R and Ramly M (2014) Fit or unfit? Perspectives of employers and university instructors of Graduates' generic skills. Procedia Soc Behav Sci 123, 315–324.

[8] Wakeham W (2016) Wakeham review of STEM degree provision and graduate employability. *Innovation and Skills: Department for Business*. [cited 2024 Sept 10]. Available from: https://assets.publishing.service.gov.uk/media/5a819e68e5274a2e8ab54f6d/ind‐16‐6‐wakeham‐review‐stem‐graduate‐employability.pdf.

[9] Kang D (2023) Prioritizing career preparation: learning achievements and extracurricular activities of undergraduate students for future success. Behav Sci (Basel) 13, 611. 37504058 10.3390/bs13070611 PMC10376569

[10] Kim J , Oh J and Rajaguru V (2022) Job‐seeking anxiety and job preparation behavior of undergraduate students. Healthcare (Basel) 10, 288. 35206902 10.3390/healthcare10020288 PMC8872297

[11] Newton G , Bettger W , Buchholz A , Kulak V and Racey M (2015) Evidence‐informed strategies for undergraduate nutrition education: a review. Appl Physiol Nutr Metab 40, 652–661. 25962618 10.1139/apnm-2014-0368

[12] van Gessel E , Picchiottino P , Doureradjam R , Nendaz M and Mèche P (2018) Interprofessional training: start with the youngest! A program for undergraduate healthcare students in Geneva, Switzerland. Med Teach 40, 595–599. 29519173 10.1080/0142159X.2018.1445207

[13] Kearney S , Perkins T and Kennedy‐Clark S (2015) Using self‐ and peer‐assessments for summative purposes: analysing the relative validity of the AASL (authentic assessment for sustainable learning) model. Assess Eval High Educ 41, 1–14.

[14] Edström K (2008) Doing course evaluation as if learning matters most. High Educ Res Dev 27, 95–106.

[15] Brown GA , Bull J and Pendlebury M (2013) Assessing Student Learning in Higher Education. 1st edn. Routledge, London.

[16] Zhao C‐M and Kuh GD (2004) Adding value: learning communities and student engagement. Research in Higher Education 45, 115–138.

[17] Hobbins J , Kerrigan B , Farjam N , Fisher A , Houston E and Ritchie K (2022) Does a classroom‐based curriculum offer authentic assessments? A strategy to uncover their prevalence and incorporate opportunities for authenticity. Assess Eval High Educ 47, 1259–1273.

[18] Chabeli M , Nolte A and Ndawo G (2021) Authentic learning: a concept analysis. Global J Health Sci 13, 12.

[19] Anderson K , Gupta S , Nava Buenfil F and Verrinder G (2022) Using experiential learning and authentic assessments to support students to become competent health promotion practitioners. Health Promot J Aust 33, 27–34. 10.1002/hpja.654 PMC9825918 35989494

[20] Fook CY and Sidhu GK (2010) Authentic assessment and pedagogical strategies in higher education. J Soc Sci 6, 153–161.

[21] Murphy V , Fox J , Freeman S and Hughes N (2017) “Keeping it real”: a review of the benefits, challenges and steps towards implementing authentic assessment. All Irel J High Educ 9, 2801.

[22] Dixon A (2022) A review of the impact of authentic assessment on the student experience & engagement in an online regulatory environment module. Ir J Acad Pract 10, 6.

[23] Wiggins G (1990) The case for authentic assessment. Pract Assess Res Eval 2, 2.

[24] Raymond JE , Homer CSE , Smith R and Gray JE (2013) Learning through authentic assessment: an evaluation of a new development in the undergraduate midwifery curriculum. Nurse Educ Pract 13, 471–476. 23140801 10.1016/j.nepr.2012.10.006

[25] Villarroel V , Boud D , Bloxham S , Bruna D and Bruna C (2020) Using principles of authentic assessment to redesign written examinations and tests. Innov Educ Teach Int 57, 38–49.

[26] Wester ER , Walsh LL , Arango‐Caro S and Callis‐Duehl KL (2021) Student engagement declines in STEM undergraduates during COVID‐19‐driven remote learning. J Microbiol Biol Educ 22, 22.1.50. 10.1128/jmbe.v22i1.2385 PMC8046661 33884093

[27] Attard C , Grootenboer P , Attard E and Laird A (2020) Affect and engagement in STEM education. In STEM Education Across the Learning Continuum: Early Childhood to Senior Secondary ( MacDonald A , Danaia L and Murphy S , eds), pp. 195–212. Springer, Singapore.

[28] Perry T (2022) Student engagement, no learning without it. Creat Educ 13, 1312–1326.

[29] Mebert L , Barnes R , Dalley J , Gawarecki L , Ghazi‐Nezami F , Shafer G , Slater J and Yezbick E (2020) Fostering student engagement through a real‐world, collaborative project across disciplines and institutions. High Educ Pedagog 5, 30–51.

[30] Shrivastava SR and Shrivastava PS (2022) Promoting active learning and student engagement in undergraduate medical education. J Med Soc 36, 39–42.

[31] Carini RM , Kuh GD and Klein SP (2006) Student engagement and student learning: testing the linkages. Res High Educ 47, 1–32.

[32] McArthur J (2023) Rethinking authentic assessment: work, well‐being, and society. High Educ (Dordr) 85, 85–101. 35194229 10.1007/s10734-022-00822-y PMC8853385

[33] Chapman E (2003) Assessing Student Engagement Rates. ERIC Digest.

[34] Wiggins BL , Eddy SL , Wener‐Fligner L , Freisem K , Grunspan DZ , Theobald EJ , Timbrook J and Crowe AJ (2017) ASPECT: a survey to assess student perspective of engagement in an active‐learning classroom. CBE Life Sci Educ 16, ar32. 28495936 10.1187/cbe.16-08-0244 PMC5459250

[35] Amerstorfer CM and Freiin von Münster‐Kistner C (2021) Student perceptions of academic engagement and student‐teacher relationships in problem‐based learning. Front Psychol 12, 713057. 34777094 10.3389/fpsyg.2021.713057 PMC8580851

[36] Herrington J , Reeves TC and Oliver R (2006) Authentic tasks online: a synergy among learner, task, and technology. Dist Educ 27, 233–247.

[37] Mercer‐Mapstone L , Dvorakova SL , Matthews KE , Abbot S , Cheng B , Felten P , Knorr K , Marquis E , Shammas R and Swaim K (2017) A systematic literature review of students as partners in higher education. Int J Stud Partner 1, 15–37.

[38] Green W (2019) Engaging “students as partners” in global learning: some possibilities and provocations. J Stud Int Educ 23, 10–29.

[39] Cook‐Sather A , Bovill C and Felten P (2014) Engaging Students as Partners in Learning and Teaching: A Guide for Faculty. John Wiley & Sons, San Francisco, CA.

[40] Setterington NA , McLean S and Woods A (2023) Design, implementation, and evaluation of students as partners interactive feedback model. Adv Physiol Educ 47, 181–193. 36633857 10.1152/advan.00182.2022 PMC10010919

[41] Geraghty JR , Young AN , Berkel TDM , Wallbruch E , Mann J , Park YS , Hirshfield LE and Hyderi A (2020) Empowering medical students as agents of curricular change: a value‐added approach to student engagement in medical education. Perspect Med Educ 9, 60–65. 31823304 10.1007/s40037-019-00547-2 PMC7012994

[42] Mello LV , Tregilgas L , Cowley G , Gupta A , Makki F , Jhutty A and Shanmugasundram A (2017) ‘Students‐as‐Partners’ scheme enhances postgraduate students' employability skills while addressing gaps in bioinformatics education. High Educ Pedagog 2, 43–57. 29098185 10.1080/23752696.2017.1339287 PMC5632996

[43] Reyes‐de‐Cózar S , Merino‐Cajaraville A and Salguero‐Pazos MR (2023) Avoiding academic burnout: academic factors that enhance university student engagement. Behav Sci (Basel) 13, 989. 38131845 10.3390/bs13120989 PMC10740539

[44] Tahmacbi B , Zare Bahramabadi M , Izadi M and Abdolhoseini H (2020) The causal relationship of job stressors, job calling and job burnout in non‐academic staff of faculties of Hamadan University of Medical Sciences. Iran J Ergon 7, 72–81.

[45] Salmela‐Aro K , Upadyaya K , Ronkainen I and Hietajärvi L (2022) Study burnout and engagement during COVID‐19 among university students: the role of demands, resources, and psychological needs. J Happiness Stud 23, 2685–2702. 35399578 10.1007/s10902-022-00518-1 PMC8974799

[46] Li J and Xue E (2023) Dynamic interaction between student learning behaviour and learning environment: meta‐analysis of student engagement and its influencing factors. Behav Sci (Basel) 13, 59. 36661631 10.3390/bs13010059 PMC9855184

[47] Boulton CA , Hughes E , Kent C , Smith JR and Williams HTP (2019) Student engagement and wellbeing over time at a higher education institution. PLoS One 14, e0225770. 31774878 10.1371/journal.pone.0225770 PMC6881016

[48] Zhoc KC , Webster BJ , King RB , Li JC and Chung TS (2019) Higher education student engagement scale (HESES): development and psychometric evidence. Res High Educ 60, 219–244.

[49] Beauchamp D and Monk J (2022) Effect of optional assessments on student engagement, learning approach, stress, and perceptions of online learning during COVID‐19. Int J High Educ 11, 87.

[50] Córdova A , Caballero‐García A , Drobnic F , Roche E and Noriega DC (2023) Influence of stress and emotions in the learning process: the example of COVID‐19 on university students: a narrative review. Healthcare (Basel) 11, 1787. 37372905 10.3390/healthcare11121787 PMC10298416

[51] Al‐Rouq F , Al‐Otaibi A , AlSaikhan A , Al‐Essa M and Al‐Mazidi S (2022) Assessing physiological and psychological factors contributing to stress among medical students: implications for health. Int J Environ Res Public Health 19, 16822. 36554703 10.3390/ijerph192416822 PMC9779130

[52] Frazier P , Gabriel A , Merians A and Lust K (2019) Understanding stress as an impediment to academic performance. J Am Coll Health 67, 562–570. 30285563 10.1080/07448481.2018.1499649

[53] Lin X‐J , Zhang C‐Y , Yang S , Hsu M‐L , Cheng H , Chen J and Yu H (2020) Stress and its association with academic performance among dental undergraduate students in Fujian, China: a cross‐sectional online questionnaire survey. BMC Med Educ 20, 181. 32493378 10.1186/s12909-020-02095-4 PMC7271445

[54] Ihab I , Brendel EBK , Law C , Van K , Martin J and Monk JM (2024) Impact of student generated exam aids on academic performance, stress and learning approach. Am J Educ Res 12, 59–69.

[55] Barbayannis G , Bandari M , Zheng X , Baquerizo H , Pecor KW and Ming X (2022) Academic stress and mental well‐being in college students: correlations, affected groups, and COVID‐19. Front Psychol 13, 886344. 35677139 10.3389/fpsyg.2022.886344 PMC9169886

[56] Cohen S , Kamarck T and Mermelstein R (1983) A global measure of perceived stress. J Health Soc Behav 24, 385–396. 6668417

[57] Monk J , Beauchamp D , Holt R and Van K (2023) Effectiveness of literature critique peer discussions to build scientific literacy skills, engagement and improve learning‐related emotions during COVID‐19‐associated online learning. Am J Educ Res 11, 303–315.

[58] Pekrun R , Goetz T , Frenzel A , Barchfeld P and Perry R (2011) Measuring emotions in students' learning and performance: the achievement emotions questionnaire (AEQ). Contemp Educ Psychol 36, 36–48.

[59] Putwain D , Sander P and Larkin D (2013) Academic self‐efficacy in study‐related skills and behaviours: relations with learning‐related emotions and academic success. Br J Educ Psychol 83, 633–650. 24175686 10.1111/j.2044-8279.2012.02084.x

[60] Dam RF and Siang TY (2021) What is design thinking and why is it so popular? [cited 2024 Sept 14]. Available from: https://www.interaction‐design.org/literature/article/what‐is‐design‐thinking‐and‐why‐is‐it‐so‐popular.

[61] Brannen S , Beauchamp D , Cartwright N , Liddle D , Tishinsky J , Newton G and Monk J (2021) Effectiveness of group work contracts to facilitate collaborative group learning and reduce anxiety in traditional face‐to‐face lecture and online distance education course formats. Int J Scholar Teach Learn 15, 5.

[62] Bieleke M , Gogol K , Goetz T , Daniels L and Pekrun R (2020) The AEQ‐S: a short version of the achievement emotions questionnaire. Contemp Educ Psychol 65, 101940.

[63] Biggs J , Kember D and Leung DYP (2001) The revised two‐factor study process questionnaire: R‐SPQ‐2F. Br J Educ Psychol 71, 133–149. 11307705 10.1348/000709901158433

[64] Siby T , Martin J , Burns J , Beauchamp D , Patil P , Pollock H , Vu J and Monk J (2023) Effects of online career training modules on undergraduate STEM Students' career readiness perceptions. Am J Educ Res 11, 214–224.

[65] Bearman M , Dawson P , Bennett S , Hall M , Molloy E , Boud D and Joughin G (2017) How university teachers design assessments: a cross‐disciplinary study. High Educ 74, 49–64.

[66] Ashford‐Rowe K , Herrington J and Brown C (2014) Establishing the critical elements that determine authentic assessment. Assess Eval High Educ 39, 205–222.

[67] Dory V , Wagner M , Cruess R , Cruess S and Young M (2023) If we assess, will they learn? Students' perspectives on the complexities of assessment‐for‐learning. Can Med Educ J 14, 94–104. 37719398 10.36834/cmej.73875 PMC10500400

[68] Lubicz‐Nawrocka T (2018) Students as partners in learning and teaching: the benefits of co‐creation of the curriculum. Int J Stud Partner 2, 47–63.

[69] Vu T and Dall'Alba G (2014) Authentic assessment for student learning: an ontological conceptualisation. Educ Philos Theory 46, 778–791.

[70] Boud D (2000) Sustainable assessment: rethinking assessment for the learning society. Stud Contin Educ 22, 151–167.

[71] Watkins D , Dahlin B and Ekholm M (2005) Awareness of the backwash effect of assessment: a phenomenographic study of the views of Hong Kong and Swedish lecturers. Instruct Sci 33, 283–309.

[72] Carpenter L , Nguyen B , Davis L and Rowland S (2022) The undergraduate research experience as a vehicle for employability development—the student participants speak. Biochem Mol Biol Educ 50, 65–74. 34668638 10.1002/bmb.21586

[73] Neely P and Tucker J (2012) Using business simulations As authentic assessment tools. Am J Business Educ 5, 449–456.

[74] Bosco AM and Ferns S (2014) Embedding of authentic assessment in work‐integrated learning curriculum. Asia‐Pac J Coop Educ 15, 281–290.

[75] Miller E and Konstantinou I (2022) Using reflective, authentic assessments to embed employability skills in higher education. J Work Appl Manage 14, 4–17.

[76] Chen G , Gully SM and Eden D (2001) Validation of a new general self‐efficacy scale. Organ Res Methods 4, 62–83.

[77] Tajima EA , Song C , Meyers MK and Maglalang JM (2022) Measuring social work competencies: comparing field instructor, classroom instructor, and student self‐assessment competency ratings. J Soc Work Educ 58, 46–62.

[78] Wagner ML , Churl Suh D and Cruz S (2011) Peer‐ and self‐grading compared to faculty grading. Am J Pharm Educ 75, 130. 21969716 10.5688/ajpe757130 PMC3175657

[79] Brown G and Harris L (2013) Student self‐assessment. Sage, Thousand Oaks, CA.

[80] Getha‐Taylor H , Hummert R , Nalbandian J and Silvia C (2013) Competency model design and assessment: findings and future directions. J Public Aff Educ 19, 141–171.

[81] Sullivan K and Hall C (1997) Introducing students to self‐assessment. Assess Eval High Educ 22, 289–305.

[82] Martinez Serrano M , O'Brien M , Roberts K and Whyte D (2017) Critical pedagogy and assessment in higher education: the ideal of ‘authenticity’ in learning. Active Learn High Educ 19, 146978741772324.

[83] Lau KL (2013) Chinese language teachers' perception and implementation of self‐regulated learning‐based instruction. Teach Teach Educ 31, 56–66.

[84] Liu XQ , Guo YX and Xu Y (2023) Risk factors and digital interventions for anxiety disorders in college students: stakeholder perspectives. World J Clin Cases 11, 1442–1457. 36926387 10.12998/wjcc.v11.i7.1442 PMC10011984

[85] Sarkar M , Overton T , Thompson C and Rayner G (2016) Graduate employability: views of Recent Science graduates and employers. Int J Innov Sci Math Educ 24, 31–48.

[86] Eldeen AIG , Abumalloh RA , George RP and Aldossary DA (2018) Evaluation of graduate students employability from employer perspective: review of the literature. Int J Eng Technol 7, 961–966.

[87] Gedye S and Beaumont E (2018) “The ability to get a job”: student understandings and definitions of employability. Educ Train 60, 406–420.

[88] Mahajan R , Gupta P and Misra R (2022) Employability skills framework: a tripartite approach. Educ Train 64, 360–379.

[89] Ledbetter ML (2012) Vision and change in undergraduate biology education: a call to action presentation to faculty for undergraduate neuroscience, July 2011. J Undergrad Neurosci Educ 11, A22–A26. 23494151 PMC3592749

[90] Kabyltaevna N , Maigeldiyeva S , Makasheva M , Saudabayeva G , Dzhanbubekova M and Nakhipbekovich K (2022) Formation of postgraduate students' professional competences through independent work. Cypriot J Educ Sci 17, 1888–1900.

[91] Rosenberg S , Heimler R and Morote ES (2012) Basic employability skills: a triangular design approach. Educ Train 54, 7–20.

[92] Moore T and Morton J (2015) The myth of job readiness? Written communication, employability, and the ‘skills gap’ in higher education. Stud High Educ 42, 1–19.

[93] Rios J , Ling G , Pugh R , Becker D and Bacall A (2020) Identifying critical 21st‐century skills for workplace success: a content analysis of job advertisements. Educ Res 49, 0013189X1989060.

[94] McGunagle D and Zizka L (2020) Employability skills for 21st‐century STEM students: the employers' perspective. High Educ Skill Work Based Learn 10, 591–606.

[95] Swafford M (2018) STEM education at the nexus of the 3‐circle model. J Agri Educ 59, 297–315.

[96] Chen L , Yoshimatsu N , Goda Y , Okubo F , Taniguchi Y , Oi M , Si K , Shimada A , Ogata H and Yamada M (2019) Direction of collaborative problem solving‐based STEM learning by learning analytics approach. Res Pract Technol Enhanc Learn 14, 24.

[97] Tan TW , Lim SJ , Khan AM and Ranganathan S (2009) A proposed minimum skill set for university graduates to meet the informatics needs and challenges of the “‐omics” era. BMC Genomics 10, S36. 19958501 10.1186/1471-2164-10-S3-S36 PMC2788390

[98] O'Neill M , Adams MP , Bandelt MJ , Chester SA , Cai W and Nadimpalli SP (2019) Cohort learning: supporting transdisciplinary communication and problem‐solving skills in graduate STEM researchers. Int J Teach Learn High Educ 31, 166–175.

[99] Iwuanyanwu PN (2020) Nature of problem‐solving skills for 21st Century STEM learners: what teachers need to know. J STEM Teach Educ 55, 4.

[100] Subramaniam M , Azmi AN and Noordin MK (2020) Problem solving skills among graduate engineers: a systematic literature review. J Comput Theor Nanosci 17, 1044–1052.

[101] Maegala NM , Nor Suhaila Y , Hasdianty A , Marini I and Hazeeq Hazwan A (2021) Assessing the problem‐solving skills among foundation level students: a STEM case study. J Phys Conf Ser 1882, 012142.

[102] Moshirabadi Z , Haghani H and Borimnejad L (2016) The perceived problem solving skill of Iranian nursing students: a cross‐sectional study. Eur Psychiatr 33, S522.

[103] Ahmady S and Shahbazi S (2020) Impact of social problem‐solving training on critical thinking and decision making of nursing students. BMC Nurs 19, 94. 33041659 10.1186/s12912-020-00487-x PMC7542695

[104] Li Y , Huang Y , Huang Y , Sun J and Wei D (2020) Study on the relationship among resilience, problem solving and coping styles of clinical nurses. J Nurs Adm 20, 328–333.

[105] Peasland EL , Scott GW , Morrell LJ and Henri DC (2024) Student employability enhancement through fieldwork: purposefully integrated or a beneficial side effect? J Geogr High Educ 48, 633–647.

[106] Scott I , Fuller I and Gaskin S (2006) Life without fieldwork: some lecturers' perceptions of geography and environmental science fieldwork. J Geogr High Educ 30, 161–171.

[107] Stokes A , Magnier K and Weaver R (2011) What is the use of fieldwork? Conceptions of students and staff in geography and geology. J Geogr High Educ 35, 121–141.

[108] Steele KJ , VanRyn VS , Stanescu CI , Rogers J and Wehrwein EA (2020) Start with the end in mind: using student career aspirations and employment data to inform curriculum design for physiology undergraduate degree programs. Adv Physiol Educ 44, 697–701. 33079563 10.1152/advan.00167.2020

[109] Connolly D , Dickinson L and Hellewell L (2023) The development of undergraduate employability skills through authentic assessment in college‐based higher education. J Learn Dev High Educ 27, 1–16.

[110] Siddique S , Ahsan A , Azizi N and Haass O (2022) Students' workplace readiness: assessment and skill‐building for graduate employability. Sustainability 14, 1749.

[111] Doménech‐Betoret F , Abellán‐Roselló L and Gómez‐Artiga A (2017) Self‐efficacy, satisfaction, and academic achievement: the mediator role of Students' expectancy‐value beliefs. Front Psychol 8, 1193. 28769839 10.3389/fpsyg.2017.01193 PMC5513915

[112] Ferla J , Valcke M and Schuyten G (2009) Student models of learning and their impact on study strategies. Stud High Educ 34, 185–202.

[113] Bandura A , Freeman WH and Lightsey R (1999) Self‐efficacy: the exercise of control. J Cogn Psychother 13, 158–166.

[114] Hayat AA , Shateri K , Amini M and Shokrpour N (2020) Relationships between academic self‐efficacy, learning‐related emotions, and metacognitive learning strategies with academic performance in medical students: a structural equation model. BMC Med Educ 20, 76. 32183804 10.1186/s12909-020-01995-9 PMC7079530

[115] Greco A , Annovazzi C , Palena N , Camussi E , Rossi G and Steca P (2021) Self‐efficacy beliefs of university students: examining factor validity and measurement invariance of the new academic self‐efficacy scale. Front Psychol 12, 498824. 35095624 10.3389/fpsyg.2021.498824 PMC8793353

[116] Meyer N , Niemand T , Davila A and Kraus S (2022) Biting the bullet: when self‐efficacy mediates the stressful effects of COVID‐19 beliefs. PLoS One 17, e0263022. 35089967 10.1371/journal.pone.0263022 PMC8797252

[117] Tahmassian K and Jalali Moghadam N (2011) Relationship between self‐efficacy and symptoms of anxiety, depression, worry and social avoidance in a normal sample of students. Iran J Psychiatry Behav Sci 5, 91–98. 24644452 PMC3939966

[118] Rohde J , Marciniak MA , Henninger M , Homan S , Paersch C , Egger ST , Seifritz E , Brown AD and Kleim B (2023) Investigating relationships among self‐efficacy, mood, and anxiety using digital technologies: randomized controlled trial. JMIR Form Res 7, e45749. 37578827 10.2196/45749 PMC10463091

[119] Schönfeld P , Brailovskaia J , Zhang XC and Margraf J (2019) Self‐efficacy as a mechanism linking daily stress to mental health in students: a three‐wave cross‐lagged study. Psychol Rep 122, 2074–2095. 30235979 10.1177/0033294118787496

[120] Asif S , Mudassar A , Shahzad TZ , Raouf M and Pervaiz T (2020) Frequency of depression, anxiety and stress among university students. Pak J Med Sci 36, 971–976. 32704273 10.12669/pjms.36.5.1873 PMC7372668

[121] Estrada‐Araoz EG , Bautista Quispe JA , Córdova‐Rojas LM , Ticona Chayña E , Mamani Coaquira H and Huaman Tomanguilla J (2023) Mental health of university students when returning to face‐to‐face classes: a cross‐sectional study. Behav Sci (Basel) 13, 438. 37366690 10.3390/bs13060438 PMC10295425

[122] Mofatteh M (2021) Risk factors associated with stress, anxiety, and depression among university undergraduate students. AIMS Public Health 8, 36–65. 33575406 10.3934/publichealth.2021004 PMC7870388

[123] Cruz J and Lopes R (2023) Self‐efficacy, stress and well‐being in the transition to higher education. Eur Psychiatr 66, S478–S479.

[124] Daoust‐Boisvert A (2022) Science communication skills as an asset across disciplines: a 10‐year case study of students' motivation patterns at Université Laval. Public Underst Sci 31, 648–659. 34736347 10.1177/09636625211051970 PMC9131417

[125] Grimshaw JM , Eccles MP , Lavis JN , Hill SJ and Squires JE (2012) Knowledge translation of research findings. Implement Sci 7, 50. 22651257 10.1186/1748-5908-7-50 PMC3462671

[126] Straus SE , Tetroe J and Graham I (2009) Defining knowledge translation. CMAJ 181, 165–168. 19620273 10.1503/cmaj.081229 PMC2717660

[127] Khoddam H , Mehrdad N , Peyrovi H , Kitson AL , Schultz TJ and Athlin AM (2014) Knowledge translation in health care: a concept analysis. Med J Islam Repub Iran 28, 98. 25664299 PMC4301207

[128] Bezanilla MJ , Fernández‐Nogueira D , Poblete M and Galindo‐Domínguez H (2019) Methodologies for teaching‐learning critical thinking in higher education: the teacher's view. Think Skills Creat 33, 100584.

[129] Papathanasiou IV , Kleisiaris CF , Fradelos EC , Kakou K and Kourkouta L (2014) Critical thinking: the development of an essential skill for nursing students. Acta Inform Med 22, 283–286. 25395733 10.5455/aim.2014.22.283-286 PMC4216424

[130] Kelp NC , McCartney M , Sarvary MA , Shaffer JF and Wolyniak MJ (2023) Developing science literacy in students and society: theory, research, and practice. J Microbiol Biol Educ 24, 1–4. 10.1128/jmbe.00058-23 PMC10443302 37614885

[131] Gormally C , Brickman P and Lutz M (2012) Developing a test of scientific literacy skills (TOSLS): measuring undergraduates' evaluation of scientific information and arguments. CBE Life Sci Educ 11, 364–377. 23222832 10.1187/cbe.12-03-0026 PMC3516792

[132] Aslam S , Delgado‐Angulo EK and Bernabé E (2017) Perceived learned skills and professional development of graduates from a master in dental public health programme. Eur J Dent Educ 21, 1–5. 10.1111/eje.12167 PMC5248589 26272511

[133] Helitzer DL , Newbill SL , Morahan PS , Magrane D , Cardinali G , Wu CC and Chang S (2014) Perceptions of skill development of participants in three national career development programs for women faculty in academic medicine. Acad Med 89, 896–903. 24871241 10.1097/ACM.0000000000000251 PMC4116611

[134] Alzaabi S , Nasaif M , Khamis AH , Otaki F , Zary N and Mascarenhas S (2021) Medical students' perception and perceived value of peer learning in undergraduate clinical skill development and assessment: mixed methods study. JMIR Med Educ 7, e25875. 34021539 10.2196/25875 PMC8317042

