# Supplementary material for: Using a ‘Students as Partners’ model to develop an authentic assessment promoting employability skills in undergraduate life science education
Source: FEBS Open Bio. 2024 Dec 5;15(3):506–22. doi: 10.1002/2211-5463.13941 (PMC11891773; doi:10.1002/2211-5463.13941)
Supplement: Supplementary file 1 — Data S1. Data extraction assignment instructions. [file FEB4-15-506-s001.docx]

**DATA EXTRACTION ASSIGNMENT INSTRUCTIONS**

**Weight:** 30% of final grade (100 possible marks)

**Submit:** Courselink Dropbox

**Part 1 - Data Extraction Table & Communication Outputs (90 marks)**

**Part 2 – Self-Assessment & Reflection (10 marks)**

**Background: Data Extraction from Scientific Literature**

When accessing scientific literature for a large project like a literature review or for smaller assignments you will need to establish a regimented and organized approach for extracting data or information from primary research articles. Researchers often use a data extraction table to organize, summarize and integrate the results from multiple primary research articles about a specific topic into one organized document. Data extraction tables are commonly included in review articles.

**Rationale:**

The Data Extraction Assignment encourages students to establish an organized method for extracting information or research findings from primary research articles. This approach can save time by avoiding the need to re-read the same research articles over and over searching for critical details, results or study design information when writing review articles or completing assignments/projects in other courses or in the future workplace.

**Relevance:**

Examples of real-world skills students will develop and practice from this assignment along with examples of careers where these skills are required are listed below.

*Examples of careers the require the use of the assignment-associated skills:*

- Laboratory Technician
- Research Associate/Assistant
- Data Analyst
- Public Health
- Academia and Teaching
- Information Technology (IT)
- Public Administration
- Scientist

*Examples of assignment-associated skills:*

- Research skills
- Data analysis and evaluation
- Critical thinking
- Extraction, organization, and integration of scientific data

**Step-by-Step Assignment Instructions and Suggested Timeline:**

This assignment is designed to be worked on throughout the semester and this approach will support your success. It is a large assignment broken down into smaller portions or steps that is intended to help students develop critical skills that are relevant for future career directions related to this course. **A *strongly suggested* timeline for completing each step in the assignment is provided below.**

**
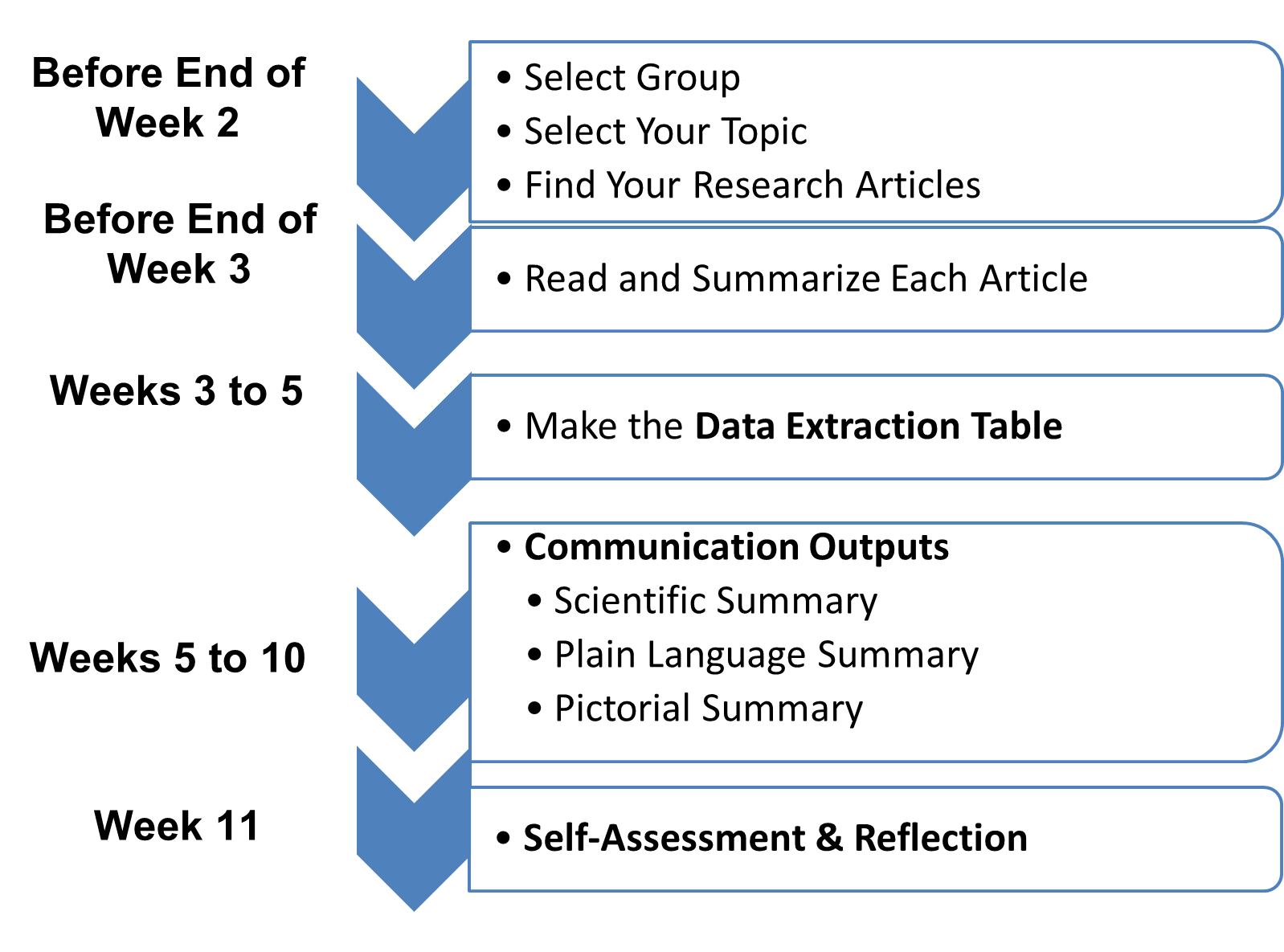
**

**Step 1: Select your Group** 🡪 ***should be completed before the end of Week 2***

You will work in **groups of** **3 students** to complete this assignment. **Students are expected to independently resolve any interpersonal disputes arising from group work**. All students working in a group will receive the same final grade on the assignment. Students interested in working in a group can use the Discussion Board Forum “*Students Looking for Assignment Group Members*” to help you connect with other students in the course.

**IMPORTANT NOTES ABOUT GROUPWORK AND GROUP DEADLINES**: All group members must sign up to be included in a group. You can sign up for a group under the “Groups” tab in the navbar in CourseLink. Make note of your group number.

**Students can select their own group between January 8^th^ (starting at 12:01am) and January 17^th^ (ending at 11:59pm).** After this time (starting on January 18^th^ at 12:01am) any students who have not signed up for a group will be randomly allocated to a group. All groups who do not have 3 group members should be aware of this deadline and reach out to new group member(s) assigned to the project. **In the workplace, colleagues (that you may or may not know) are randomly assigned to work together, so this element of the assignment simulates a critical workplace experience**. Students are encouraged to view this as an opportunity to work with new individuals that bring unique perspectives and important contributions to the project.

If you are a student assigned to an existing group be aware that the other group members may have already selected a topic and you will need to join in and contribute. This may not be your preferred topic choice, however, **part of simulating the workplace experience is compromising and adapting to and contributing to projects that are already in progress**.

At the start of the group work process, each group will collectively make decisions regarding if all group members will work collaboratively on all elements of the assignment OR if individual group members will be responsible for LEADING each communication output. **Leading a communication output means writing a first draft, all group members are responsible of the final submission of ALL ELEMENTS of the project.** All group members are responsible for:

- Finding and summarizing the research articles into the data extraction table
- The content in the final data extraction table
- Editing and finalizing the content in each communication output

**Step 2: Select your Topic** 🡪 ***should be completed before the end of Week 2***

Choose any chronic disease or health condition AND any dietary component, bioactive or nutrient that has been shown to modulate the disease of health condition of interest. The dietary component can drive the development of the condition or alleviate/attenuate the condition. Follow your interests.

**OPTIONAL FEEDBACK** – you can post your topic idea to the “*Assignment Topic Feedback*” Discussion Board and the TA or Instructor will reply with suggestions to refine your topic to help you or to give their approval of your topic. This is not necessary, but some students may find it helpful. Please note that due to the number of students in the course you will receive a response within 72 hours of posting your topic idea.

**Step 3: Access the Scientific Literature & Select Research Articles** 🡪 ***should be completed during Weeks 1-3***

Find, read and summarize 5 primary research articles all related to your topic.

You need to ensure that these research articles are related, so you will likely need to read more than the 5 required primary research articles in order to find good articles on a related topic. These papers do not need to all use the same study design BUT they should be measuring a similar mechanistic target and/or clinical outcome. Since there will be a mechanistic focus in this assignment observational studies are not permitted since they only show an association.

**Step 4: Make a Data Extraction Table (30 marks)** 🡪 ***should be completed during Weeks 3-5***

***The Data Extraction Table is included in Part 1 of the Submitted Assignment***

Each student or group must figure out the best approach for extracting, organizing and integrating information from scientific literature. Two types of approaches for constructing the table that students may use are:

1. Directly going from reading the articles to making the data extraction table. The challenge with this approach is that key information that should be a column or category in the data extraction table may be overlooked.
2. Making a 1-page point form summary of each research article FIRST (i.e., a data extraction page for each article), and then using this content to integrate the study results together into a final data extraction table. This approach sounds like more work, but it is a more organized approach that is going from the larger more complicated summary of your primary research articles to a concise table.

Your data extraction table should focus on only 1-2 main outcomes/results. Your research articles will have measured multiple outcomes, but your assignment will only focus on 1-2 main outcomes that are common to all 5 articles for the focus of your assignment. These results/outcomes should center upon a mechanism of action or a critical clinical outcome. **Example:** If you focus on tumor size as a clinical outcome, you MUST discuss possible mechanisms of action that underlie this outcome in your scientific summary, for instance, how is your dietary component *hypothesized* to mechanistically reduce tumor size, such as reducing cell proliferation (e.g., reducing Ki67 expression) or stimulating tumor cell apoptosis (e.g., increasing TRAIL expression).

**Data Extraction Table Formatting**

The data extraction table will likely NOT fit on one page. You may reduce the font size to 10-point font in the table and may find LANDSCAPE ORIENTATION helpful. Formatting requirements for the data extraction table are flexible, however, column headings must be bolded. If your table extends onto a second or third page, have the page break occur between research articles (so that all information from a single research article appears on the same page). Please repeat the column headings on each page if your table is more than 1 page. Refer to the assignment exemplars. You may use the point form in the table.

**Suggestions for Column Headings in the Data Extraction Table**

**Providing the research article citation in the data extraction table is mandatory**.

The other column headings are up to each student/group to determine. Some examples of the suggested types of column headings that should be included in a data extraction table are listed below. These suggested headings include important information; however, you may edit/refine these suggestions to align with your topic and selected research articles. ***Students* *are encouraged to add any other relevant column headings as needed based on their topic.***

- **Type of study/study design** Experimental (cell culture studies, clinical trials, RCTs, etc. (Cannot use observational studies!)
- **Study population (and include relevant details)**
  Human study (age, gender, sample size, health status: e.g., healthy, obese, T2D, etc.)

Animal study (species, age, sample size, strain, etc.)
 Cell Culture/in vitro study (cell line, number of culture wells or replicates per treatment, etc.)

- **Duration of study or intervention**
- **Dose and timing of the intervention**
- **Key methods used to generate the results** (e.g., obesity status could be assessed using different measurements such as BMI, waist circumference, body weight, body composition scan, blood biomarker(s), etc.)
- **Results/Endpoints:** *have a separate bolded heading for each main result/endpoint* (e.g., tumor number, serum insulin concentration, etc.). It is important to note how each endpoint changed in the treatment versus the control group (an increase, decrease or no change/no effect). It is important to write down the results/endpoints that relate directly to your topic, as some studies will measure endpoints that might not be relevant.

***Steps 5 - 8 are Types of Communication Outputs Generated from the Data Extraction Table and should be completed between Weeks 5-10 of the semester***

**Step 5: Scientific Summary of the Content in the Data Extraction Table (30 marks)**

***The Scientific Summary is included in Part 1 of the Submitted Assignment***

This is a targeted form of communication for a scientific audience. Write a 2-page (single-spaced,12-point font) summary of the research findings from the data extraction table you generated. This Scientific Summary will start with a short introductory paragraph of your topic that discusses key information about the disease/health condition and the dietary component you selected. This introductory paragraph should be supported with citations from scientific literature that are NOT the 4 or 5 primary articles summarized in your data extraction table. These references should be scientific journal articles and websites may only be used for presenting disease/health condition statistics (e.g., prevalence of breast cancer in Canadian women cited from the Canadian Cancer Society website). **There should be a bolded sentence or two at the end of the introductory paragraph that outlines what the remainder of your Scientific Summary paper will discuss (this is also the focus of your data extraction table)**. The middle of your summary paper should discuss and elaborate upon the content of your data extraction table. Research findings should be integrated. Strengths and limitations of the study designs that influence the interpretation of the results should be discussed. Additionally, the mechanism of action through which your selected dietary bioactive or dietary component influences the disease pathophysiology or phenotype should be explained.

- This can be a mechanism of action determined from the study results. E.g., Diet component X activates caspase-3 stimulating apoptosis and liver tumor cell growth is reduced.
- This can be a *putative* or *hypothesized* mechanism of action. E.g., Diet component X reduces liver tumor cell growth, which is hypothesized to be through stimulation of apoptotic signaling. Monk et al. 2022 (a paper that’s not in your data extraction table) showed that diet component X activates caspase-3 and stimulates apoptosis in prostate tumor cells and a similar mechanism could underlie the reduction in tumor cell growth observed in the studies summarized in the data extraction table. This potential mechanism of action could be tested in a follow-up study…(now you explain your ideas for this follow-up study!) This should help lead you to the conclusion.

The concluding paragraph should identify knowledge gaps and what type of future research is required to move this research front forward (e.g., to resolve the conflicting research findings that you identified in your data extraction table and scientific summary OR to test a mechanism of action observed in animal studies in a human clinical population, etc.).

In-text citations should be numbered (to save space in the 2-page limit) and references are NOT included in the 2-page limit. References should be formatted in APA style:


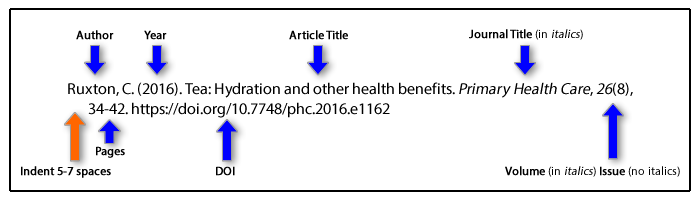


**Step 6: Plain Language Summary of the Content in the Data Extraction Table (15 marks)**

***The Plain Language Summary is included in Part 1 of the Submitted Assignment***

This is a *targeted form of communication for a general or non-scientific audience.* It is a necessary form of communication/knowledge translation; however, it can be challenging to accurately convey scientific information to a general audience in a form that they can understand. Write a plain language summary discussing the main findings reported in your data extraction table. This should be written in clear, easy-to-understand language where the scientific content could be understood by someone without a science background or education. The plain language summary should be approximately 0.5 page single-spaced (400 words maximum) using 12-point Times New Roman font. Typically, plain language summaries do not include in-text citations, however, you should provide a short reference list at the bottom of the plain language summary citing the papers that appear in your data extraction table (using APA format). References are not included in the word/space limit. This is similar to references provided to support a scientific article summary or scientific information on reputable websites or the general public.

**Step 7: Pictorial Summary of the Content in the Data Extraction Table (15 marks)**

***The Pictorial Summary is included in Part 1 of the Submitted Assignment***

Create a pictorial representation of the research findings and mechanism of action outlined in your data extraction table. This is NOT an infographic; what you create is smaller in scope and content. The pictorial representation is similar to what would be required for a graphical abstract in scientific papers or in communication/education bulletins communicated to the public (either in print or online). The aim of this pictorial representation is to **integrate the content presented in the data extraction table about your topic and translate this into a visual summary**. This may include a combination of text and diagrams/images; however, text content should be minimized and used in the form of titles, legends/captions, and/or short annotations. You will NOT be graded on the specific design and colour used because this is subjective, however, your graphical abstract should be visually appealing (i.e., NOT monochromatic). Making a pictorial representation represents an opportunity to be creative and practice communicating scientific concepts using a diagram that you create. The content of your pictorial representation should focus on the accuracy and clarity of scientific concept depicted in your data extraction table, and you will be assessed on how well this concept(s) are captured in the pictorial representation. References are not needed, as they are already provided in the data extraction table. The pictorial representation should be the size of one power point slide and submitted as a PDF in your assignment. There are no other formatting requirements – you have freedom to use your creativity. The exemplars provided to inspire your creative thinking; they are NOT templates.

**Step 8: Self-Assessment & Reflection (10 marks) *🡪 Part 2 of the Assignment (week 11 of the semester)***

**Must be completed INDEPENDENTLY by EVERY STUDENT even if you worked in a group.** *Self-assessment exemplars are shown; however, reflection exemplars are not shown as these are unique and personal for each student. Refer to the rubric for clarification about reflection expectations*

Conducting employee performance quality improvement exercises are common in most workplaces. This is a type of performance-based self-assessment, which provides an opportunity for individuals to identify areas or skills that require further improvement/development and areas or skills that are satisfactory or exemplary. Every student will be asked to complete their own reflection and self-assessment of their assignment. These files will be submitted to the Dropbox separately from the other components of the data extraction assignment.

The **self-assessment** will consist of EACH student’s critical evaluation and grading of your Data Extraction Assignment using the assignment component rubrics. If you worked in a group each individual student is still responsible for the entire assignment, therefore, it is important to work collaboratively. You will highlight in any colour of your choice on the rubric which category your work falls into (e.g. “Needs Improvement”, “Meets Expectations”, “Exceeds Expectations”). Highlight the specific criteria text within each rubric column that applies to your work (if working as an individual) or your groups work (if you worked in a group). Remember, if you worked in a group to complete the assignment each group member is responsible for the entire finished product. Assign a numerical grade to each component of the Data Extraction Assignment and a total final grade using the **Self-Assessment Grading Template** posted in CourseLink. **You are expected to be critical, reflective and honest**. If you determine that your work “exceeds expectations” as outlined in the rubric then you should assign yourself that corresponding numerical grade. If you determine that elements of your work “need improvement” then identify what those elements are by highlighting the corresponding text in the rubric and assign yourself a numerical grade accordingly. There is no judgement on the part of the Instructor or TA about the numerical grade that you believe reflects your final assignment quality. This may feel awkward or uncomfortable (particularly if the grade you assign yourself is very high or very low) but the process is important, and the rubrics will guide your grading decisions in the self-assessment of your work. What is important is that each student critically evaluates their own work and practices this type of self-assessment. The reflection is where you can provide some context for the self-assessed grade you provided by following the reflection question prompts.

The **reflection** will consist of each student’s response to the reflection question prompts. Your reflection should be written in the first person and be a minimum length of 2 pages of text (single-spaced, 12 point font) for all questions combined, however, it is possible that your responses will be longer and this is okay. Use the amount of space you require to actually reflect on your assignment and learning experience. Your reflection will be graded based on the quality of your answers and providing a response to each reflective question. More detailed guidelines for the assessment of your assignment reflection are provided in the Self-Assessment Grading Template. You will provide an honest assessment of your effort applied to the assignment in your reflection. Full marks for each reflection criteria will expect students to explain the *“how”* or *“why”* they came to the conclusions or perspectives that are expressed in the reflection. This is the difference between *“awareness”* and *“reflection”*, as outlined in the reflection rubric. Note – *employers frequently also require a written reflection as part of a self-assessment or annual performance assessment, which is why we are practicing these skills in our course.*

All components of the Data Extraction Assignment should be submitted to the Dropbox in CourseLink. There are 2 files that will be submitted (Part 1 and Part 2) on different due dates (see check list below):

**Part 1: Data Extraction Table and Communication Outputs** Components Merged into One PDF File per GROUP (90 marks total)

- **Data Extraction Table** (30 marks)
- **Scientific Summary** (30 marks)
- **Plain Language Summary** (15 marks)
- **Pictorial Summary** (15 marks)

**Part 2: Reflection and Self-Assessment** Components Merged into One PDF File per STUDENT (10 marks total)

- **Reflection**
- **Self-Assessment** **Grading Template**

**NOTES about Self-Assessment/Grading**

The rubrics posted on CourseLink for each component within the Data Extraction Assignment are divided into “below”, “meets” or “exceeds” expectations. If ANY criteria are in the “below expectations” category then the assessment for that criterion overall is still below expectations, despite other elements within that criteria that may be well done (see example rubric below “Critical Insight of Research” criteria). To get 100% on the assignment would require a student to exceed expectations for all criteria. **All rubrics are posted on CourseLink.**
