# Supplementary material for: Using a ‘Students as Partners’ model to develop an authentic assessment promoting employability skills in undergraduate life science education
Source: FEBS Open Bio. 2024 Dec 5;15(3):506–22. doi: 10.1002/2211-5463.13941 (PMC11891773; doi:10.1002/2211-5463.13941)
Supplement: Supplementary file 2 — Data S2. Data extraction assignment self‐assessment template, rubrics and reflection questions. [file FEB4-15-506-s002.docx]

**Data Extraction Assignment Self-Assessment Template**

Use the rubrics (attached) to provide a honest critical assessment of your Data Extraction Assignment. Fill in the table below with your numerical grade for each rubric criteria and indicate if your assessment of your assignment is *below*, *meets*, or *exceeds* rubric expectations. On the attached rubics (that you will use to assess your work) highlight the criteria that applies to your assessment using any colour in the highlighting tool. Refer to the student exemplars to clarify the instructions as needed. NOTE: You cannot earn full marks for any criteria unless your work exceeds expectations.

**Name:**

**Student Number:**

| **Criteria** | **Below, Meets, or Exceeds Expectations** | **Grade** |
| --- | --- | --- |
| **Part 1: Data Extraction Table (out of 30 marks)** | | |
| **Topic is Appropriate** (2 marks) |  |  |
| **Selected Papers are Relevant and Appropriate for the Topic** (4 marks) |  |  |
| **Appropriate Column Headings in the Table** (5 marks) |  |  |
| **Level of Detail** (15 marks)  Need to Consider the following (5 marks for each):  *Level of Detail*  *Concise Text*  *Accurate Text* |  |  |
| **Formatting/Organization** (4 marks) |  |  |
| **Total Grade for Data Extraction Table** |  |  |
| **Part 2: Scientific Summary (out of 30 marks)** | | |
| **Introduction** (2.5 marks) |  |  |
| **Accurate reporting of research findings** (5 marks) |  |  |
| **Critical insight of research** (15 marks**)** |  |  |
| **Conclusion** (5 marks) |  |  |
| **Writing Style** (2.5 marks) |  |  |
| **Total Grade for Scientific Summary** |  |  |
| **Part 3: Plain Language Summary (out of 15 marks)** | | |
| **Accuracy** (3 marks) |  |  |
| **Focus**(3 marks) |  |  |
| **Simple language** a**ppropriate for a non-scientific audience/reader**  (4 marks) |  |  |
| **Organization** (3 marks) |  |  |
| **Formatting** (2 marks) |  |  |
| **Total Grade of Plain Language Summary** |  |  |
| **Part 4: Pictorial Summary (out of 15 marks)** | | |
| **Content** (5 marks) |  |  |
| **Mechanism of Action** (5 marks) |  |  |
| **Attractiveness, Graphics & Text** (3 marks) |  |  |
| **Formatting** (2 marks) |  |  |
| **Total Grade for Pictorial Summary** |  |  |
|  |  |  |
| **Total Self-Assessed Grade (out of 90 marks)** |  |  |

**Data Extraction Assignment Component Rubrics**

**Data Extraction Table Rubric (30 marks)**

| **Criteria** | **Need Improvement to Reach Expectations** | **Meets Expectations** | **Exceeds Expectations** |
| --- | --- | --- | --- |
| **Topic is Appropriate**  **(2 marks)** | The topic selected is not focused enough to align with the selected papers. Does not focus on one dietary component and one disease/condition. | Topic is appropriate and sufficiently focused to be able to select the number of required studies in the table that are related to each other. |  |
| **Selected Papers are Relevant and Appropriate for Your Topic (4 marks)** | Insufficient number of papers included in the table.  Observational studies are included.  The selected papers are not related to the topic. | All papers are relevant to the selected topic so that they can be compared and contrasted in the data extraction table.  No use of observational studies. |  |
| **Appropriate Column Headings in the Table (5 marks)**  Your table should use appropriate headings for the information that’s required to interpret the results, compare between studies in the table and for the reader to comprehend the studies. | Critical information that should have been summarized in a separate heading in the table is missing. | Critical information is provided in table headings but additional information that would support interpretation of the results and comprehension of the studies is omitted OR additional irrelevant headings are included. | All headings in the table are relevant and required to interpret the results and for the reader to comprehend the studies. |
| **Level of Detail (15 marks)**  The level of detail in your table should allow the reader to comprehend the studies and to compare outcomes between studies.  Text in the table is concise and accurate. No copied text directly from papers. | Insufficient amount of information is provided. Point form summary of the information is either too detailed containing irrelevant information OR cannot be interpreted because details are missing for one or more than one studies.  No use of point form or point form cannot be understood by the reader and is incomplete. Some information is inaccurate.  Some information appears to be cut and paste text from papers. | Sufficient level of detail is provided in most places.  There are very few instances where unnecessary additional information is provided, or minimal information is provided so that details are incomplete.  Attempts to be concise with communication of study details are evident. Sentences are in point form, in the students’ own words and can be comprehended by readers.  All information is accurate | Text is concise and accurate. Only relevant information is provided with no instances of unnecessary details about the studies that deviate from the topic of the data extraction table. |
| **Formatting/Organization (4 marks)** | Does not follow formatting and organization instructions. | Follows the formatting instructions, with some errors in spelling, table headings, or in the organization of the table. | Follows the formatting instructions and table headings are presented in a logical and organized manner reading the table left to right. |

**Scientific Summary Rubric (30 marks)**

| **Criteria** | **Below Expectations** | **Meets Expectations** | **Exceeds Expectations** |
| --- | --- | --- | --- |
| **Introduction.**  Your paper should provide a general background about your topic. The topic should be clearly stated.  **(2.5 marks)** | Topic is introduced, but critical information needed to understand the data extraction table contents is not provided.    The relevance of the topic or specific focus of the selected topic is not clearly explained. | Topic is introduced with appropriate context/background information.    The topic for the data extraction table is clearly explained. | Appropriate background information is provided in addition to explaining why the topic is relevant or important.  The background information is provided in an engaging and organized manner that leads to the topic focus of the data extraction table. |
| **Accurate reporting of research findings.**  Your paper should accurately cover the key elements of the studies in your table. **(5 marks)** | Unnecessary details included, or significant details omitted, which detract from a central message or confuse the reader.  There is no integration of research findings. | Key aspects of research (eg: study design, results) are presented to give an accurate understanding of the findings. There is some integration of research findings. | Effectively highlights and prioritizes key aspects of each study.  Articles results are integrated (eg: compared, contrasted) in a natural flow. |
| **Critical insight of research**.  Your paper should consider the strengths and limitations of the reviewed articles, which should be integrated into the body of the scientific summary and show integration between studies. **(15 marks)** | Research may be presented at face value, or with only superficial or minimal discussion of limitations. Does not address study strengths and limitations that influence the interpretation of findings or impact the ability to directly compare results between studies.  Conflicting findings between studies are not addressed.  Limited or missing mechanism of action and knowledge gaps/future directions. | Relevant strengths and limitations of the studies are presented, but they may be handled separately or be less insightful than stronger papers written by peers in the class.  Explanation of how studies strengths and limitations impact the interpretation of the research findings is provided most of the time.  Identification of knowledge gaps/future directions and discussion of a mechanism of action are incomplete. | Original and critical discussion of the research studies as a set.    Anticipates potential objections, and guides reader to understand the balance between strengths and limitations of studies.  Knowledge gaps and future directions are clearly explained and connected to the research study findings. A mechanism of action (either directly resulting from the study results or hypothesized based on other literature connected to the studies in the data extraction table) is clearly explained. |
| **Conclusion.**  Should identify knowledge gaps and propose what future research is required to move the research front related to your topic forward. The conclusions you draw should be based on the totality of evidence presented in your data extraction table. It should also leave the reader with a “take home message”  **(5 marks)** | Conclusion is often overstated, or undersells the research presented in the table.  Knowledge gaps are not clearly identified or do not consider the totality of evidence from the studies in the table.    Relevance of the information for a general population is unclear, on unimaginative. | Conclusion is appropriate given the evidence discussed throughout the paper and table.  Knowledge gaps are identified and future research to address the gaps is presented but could be more clearly explained.    The significance of the new information for the general population is clear. | Conclusion is accurate and effectively incorporates research findings, original insights and contextual factors.  Knowledge gaps are clearly explained and future research to address these gaps is logical, scientifically accurate and appropriate for a 4^th^ year student.    The significance of the new information for the general population/or segment of the population impacted is clear and original. |
| **Writing Style.**  Your paper should be written in an engaging style to capture the reader’s interest.  **(2.5 marks)** | Writing style may be overtly technical or overly simplified or make use of flowery language. Organization is hard to follow.   Proofreading is required. Does not follow the formatting requirements and referencing style.  Inappropriate use of references (e.g., over-reliance on papers in the table or 1-2 references only for background information) | Clear and engaging writing style that is appropriate for a scientific audience.  Clear organization and flow. Minimal errors in grammar. References and formatting are correct.  Appropriate references are used for background information (primary literature, recent publications (at least 50% within the last 5 years), not relying exclusively on review articles) | Clear, concise and engaging writing style.  All sections of the paper have a purpose.  Logical structure and well edited with no errors in grammar.  Appropriate references used in the background information. |

**Plain Language Summary Rubric (15 marks)**

| **Criteria** | | **Below Expectations** | **Meets Expectations** | **Exceeds Expectations** |
| --- | --- | --- | --- | --- |
| **Accuracy**  (3 marks) | **Accurate summary of the topic and results in the data extraction table** | The summary uses vague or inaccurate language / descriptions in many places throughout | The summary is accurate; language / descriptions are vague or inaccurate in only one or two places and these do not interfere with understanding | The summary is accurate, using specific, picturable language to describe the activities and findings of the studies |
| **Focus**  (3 marks) | **The summary focuses on the key points that a reader needs to know** | The summary captures very few relevant points and misses key ideas.  Some irrelevant details are included | The summary captures most of the relevant ideas, including one or two irrelevant details that create only minor distractions | The summary focuses on all major points of the article and does not include unimportant details. |
| **Simple language**  **Appropriate for a non-scientific audience**  (4 marks) | **The summary simplifies the language of the data extraction table and topic without losing the scientific meaning** | The summary uses everyday language in some places, but often introduces errors with word substitutions or uses technical terms that the reader might not know | The summary consistently uses everyday language of the intended reader.  Occasionally uses creative and effective ways to translate complex ideas. Occasionally there are technical terms or word choices that the reader might not know | The summary consistently uses everyday language of the intended reader. Consistently uses creative and effective ways to translate complex ideas |
|  | **Sentences use *active voice* and are constructed to minimize possible misinterpretation** | Few sentences use active voice, and in several places, sentences are difficult to understand | Most sentences use active voice and are easy to understand | All sentences use active voice and are easy to understand |
| **Organization**  (3 marks) | **Content is well structured with the topic introduced and an easily understood progression of information presented in the table** | The topic is introduced but the flow of the writing is not easy to follow or presented in a logical order | Well organized, with minor exceptions | The summary is well organized; ideas flow smoothly from one to the next |
| **Formatting**  (2 marks) | **Meets the instructions provided about length, referencing and content** | Does not follow the instructions | Accurately and completely follows the instructions |  |

**Pictorial Summary Rubric (15 marks)**

| **Category** | **Below Expectations** | **Meets Expectations** | **Exceeds Expectations** |
| --- | --- | --- | --- |
| **Content**  **(5 marks)** | Scientific content is minimal. There are factual errors or critical details required to understand the topic are missing. No or minimal integration of study results in the presentation.  Conflicting findings between studies (if they exist) are not addressed in any way. | Includes the necessary content to understand the topic. All information is accurate. Study results from the table are integrated together.  Any conflicting findings between studies are acknowledged. | All content required to understand the topic is included. Study results are integrated to draw conclusions.  Any conflicting findings between studies are acknowledged appropriately to highlight an identified knowledge gap related to your topic. |
| **Mechanism of Action**  **(5 marks)** | Mechanism of action (demonstrated by the study findings) or putative/hypothesized mechanism of action is not shown.  The mechanism of action is not accurate or misinterpreted. | A mechanism of action provided in some capacity and it is accurate. | The mechanism of action is provided in a clear and accurate way. Putative mechanisms of action are clearly identified and the connection to the studies in the data extraction table is made. |
| **Attractiveness, Graphics & Text (3 marks)** | Use of graphics are minimal, and the majority of information is presented using text boxes. Minimal use of colour or font in the presentation. | Balance between graphics and text boxes. Text is used to highlight critical details. Appealing use of colour | Makes excellent use of font, colour and graphics to enhance the presentation and is visually appealing. More use of graphics versus text. Text is only used only to highlight critical details. |
| **Formatting**  **(2 marks)** | Does not follow the formatting requirements in the assignment instructions. There are some misspellings. | There are minimal misspellings. All formatting requirements are correctly followed |  |

**Data Extraction Assignment Student Reflection Questions**

1. Identify and discuss the relevance of a STRENGTH you’ve learned about yourself (attribute or skill) as a result of completing the data extraction assignment.
2. Identify and discuss the relevance of a WEAKNESS (or AREA FOR IMPROVEMENT) you’ve learned about yourself (attribute or skill) as a result of completing the data extraction assignment.
3. Discuss what you found the most challenging about the assignment.
4. Explain how the process of completing the assignment contributed to the development of a new perspective OR challenged your point of view OR introduced you to a new skill.
5. Discuss the skills you developed or enhanced while completing the assignment that you feel are valuable in your future career (Discuss a minimum of 3 skills. If you don’t think you developed 3 skills discuss and reflect upon why).
6. Explain how you will use what you learned or the skills you developed/practiced while completing the data extraction assignment in the workplace. Make sure you identify the target workplace in your response.
7. If you had the opportunity to repeat this assignment again, what would you do differently?
8. Explain how you think this assignment helped prepare you for activities in the workplace.
